# Initial characterization of a *Syap1* knock-out mouse and distribution of Syap1 in mouse brain and cultured motoneurons

**DOI:** 10.1007/s00418-016-1457-0

**Published:** 2016-06-25

**Authors:** Dominique Schmitt, Natalia Funk, Robert Blum, Esther Asan, Lill Andersen, Thomas Rülicke, Michael Sendtner, Erich Buchner

**Affiliations:** 1 Institute of Clinical Neurobiology, University of Würzburg, Versbacher Str. 5, 97078 Würzburg, Germany; 2 Institute of Anatomy and Cell Biology, University of Würzburg, 97070 Würzburg, Germany; 3 Institute of Laboratory Animal Science, University of Veterinary Medicine Vienna, 1210 Vienna, Austria

**Keywords:** Brain, BSTA, Viability, Syap1 localization, Glutamatergic synapses, PKB/Akt phosphorylation

## Abstract

Synapse-associated protein 1 (Syap1/BSTA) is the mammalian homologue of Sap47 (synapse-associated protein of 47 kDa) in *Drosophila*. *Sap47* null mutant larvae show reduced short-term synaptic plasticity and a defect in associative behavioral plasticity. In cultured adipocytes, Syap1 functions as part of a complex that phosphorylates protein kinase Bα/Akt1 (Akt1) at Ser^473^ and promotes differentiation. The role of Syap1 in the vertebrate nervous system is unknown. Here, we generated a *Syap1* knock-out mouse and show that lack of Syap1 is compatible with viability and fertility. Adult knock-out mice show no overt defects in brain morphology. In wild-type brain, Syap1 is found widely distributed in synaptic neuropil, notably in regions rich in glutamatergic synapses, but also in perinuclear structures associated with the Golgi apparatus of specific groups of neuronal cell bodies. In cultured motoneurons, Syap1 is located in axons and growth cones and is enriched in a perinuclear region partially overlapping with Golgi markers. We studied in detail the influence of *Syap1* knockdown and knockout on structure and development of these cells. Importantly, *Syap1* knockout does not affect motoneuron survival or axon growth. Unexpectedly, neither knockdown nor knockout of *Syap1* in cultured motoneurons is associated with reduced Ser^473^ or Thr^308^ phosphorylation of Akt. Our findings demonstrate a widespread expression of Syap1 in the mouse central nervous system with regionally specific distribution patterns as illustrated in particular for olfactory bulb, hippocampus, and cerebellum.

**Electronic supplementary material:**

The online version of this article (doi:10.1007/s00418-016-1457-0) contains supplementary material, which is available to authorized users.

## Introduction

The synapse-associated protein of 47 kDa (Sap47) in *Drosophila* represents the founding member of a family of synapse-associated proteins with a BSD domain. Sap47 has originally been identified by a monoclonal antibody that binds to most neuropil regions of larval and adult brains (Reichmuth et al. 1995; Hofbauer et al. 2009). The superfamily of proteins containing a BSD domain includes functionally diverse proteins such as **B**TF2-like transcription factors, **S**ap47 homologues, and **D**OS2-like proteins involved in ubiquitin metabolism and control of single-copy DNA replication (Doerks et al. 2002). In glutamatergic larval motoneurons of *Drosophila*, the Sap47 protein is concentrated in presynaptic boutons in close proximity to synaptic vesicles. Basic synaptic transmission is normal in *Sap47* null mutants, but current clamp recordings at larval neuromuscular junctions reveal enhanced synaptic depression during high-frequency stimulation, indicating a defect in short-term synaptic plasticity. At the behavioral level, *Sap47* null mutant larvae show a ~50 % reduction in the ability to learn and/or remember the association of an odorant with a rewarding tastant (Funk et al. 2004; Saumweber et al. 2011).

The mammalian homologue of Sap47 termed Syap1 is widely expressed as its mRNA is detected in most human tissues (Chang et al. 2001). It has been shown to be differentially regulated by tamoxifen in breast cancer cells (Al-Dhaheri et al. 2006). Recently, Syap1/BSTA (**B**SD domain-containing **s**ignal **t**ransducer and **A**kt interactor) was shown to play an essential role in adipocyte differentiation from embryonic stem cells by promoting phosphorylation of Akt1 at Ser^473^ after growth factor stimulation which results in suppressed expression of the gene for the FoxC2 transcription factor. It was demonstrated that in dividing cells, the BSD domain is essential for the interaction between Syap1 and Akt1 which in turn appears to depend on mTORC2-mediated Syap1/BSTA phosphorylation (Yao et al. 2013). These results raise the question whether Syap1/BSTA or Sap47 deficiencies could also modify Akt signaling in differentiated neurons, which could offer a molecular explanation for the observed plasticity defects in *Sap47* mutants of *Drosophila*. In vertebrates, the three isoforms of serine/threonine kinase B (PKB/Akt) play important roles in nervous system-related functions such as neuronal differentiation, neurite growth, neuronal survival, and regulation of transcription and protein synthesis as well as neuronal maintenance and plasticity. Thus, a defect in Akt regulation caused by Syap1/Sap47 deficiency would certainly be compatible with altered synaptic plasticity and behavior as observed in *Sap47* null mutant flies.

No information on Syap1 function in the mammalian nervous system is presently available. In both humans and mice, the *Syap1* gene is located on the X-chromosome. In a mouse mutational screen of X-chromosomal genes, a gene-trap insertion leading to a hemizygous *Syap1* mutant embryo at stage E9.5 showed no obvious morphological alterations and was therefore not further investigated (Cox et al. 2010). Here, we established a knock-out mouse line from an embryonic stem cell line with a targeted mutation of *Syap1* and use knock-out animals of the first four generations as negative controls to provide an initial immunochemical characterization of the distribution of Syap1 in brain tissue and cultured embryonic primary motoneurons. We observe that *Syap1* knockout does not cause obvious morphological defects in young mice or gross structural changes in brain morphology. Immunoreactivity in wild-type mouse brain sections detected with a polyclonal antiserum generated against human Syap1 indicates that the protein is widely expressed in virtually all brain areas with strong signals in perikarya of subpopulations of neurons and in neuropil regions particularly rich in glutamatergic synapses. After 7 days in culture, *Syap1* knock-out motoneurons show normal axon length and survival rate. Neither knockdown nor knockout of *Syap1* in cultured motoneurons was associated with altered activation of Akt. Our results indicate that organismal function of Syap1 appears to be more subtle than expected considering its requirement for adipocyte differentiation, and we have no evidence that its molecular function in cultured motoneurons involves the activation of the PI3K/Akt pathway.

## Materials and methods

### Animals and ethics statement

C57BL/6J and CD1 mice were kept at the animal facilities of the Institute of Clinical Neurobiology at the University Hospital of Würzburg providing controlled conditions such as ad libitum food and water supply, at 20–22 °C, 55–65 % humidity, and a 12/12-h light/dark cycle. Specific pathogen-free quality of the animals was frequently tested and confirmed according to FELASA recommendations. Each experiment was performed strictly following the regulations on animal protection of the German federal law, the Association for Assessment and Accreditation of Laboratory Animal Care and of the University of Würzburg, in agreement with, under control of, and with the approval of the local veterinary authority and Committee on the Ethics of Animal Experiments, i.e., Regierung von Unterfranken and Veterinaeramt der Stadt Würzburg (License numbers 566/200-244/13 and 55.2-2531.01-08/14). Parts of the present study were discussed and approved by the institutional ethics committee of the University of Veterinary Medicine Vienna, and an animal experiment license was granted under BMWF-68.205/0023-II/3b/2014 (Austrian Federal Ministry of Science and Research).

### Generation of *Syap1* knock-out mice

ES cell clone HEPD0680_2_C02 with a targeted *Syap1* locus was obtained from the “The European Conditional Mouse Mutagenesis Project” (EUCOMM, Munich). The targeted mutation was induced according to the general strategy of EUCOMM, resulting in a “knockout-first” lacZ-reporter-tagged *Syap1*^*tm1a*^ insertion allele with conditional potential. The insertion in intron 3 includes the mouse En2 splice acceptor to generate a null allele through splicing to a *lacZ* trapping element with an SV40 polyadenylation signal (Skarnes et al. 2011).

**Fig. 1:**
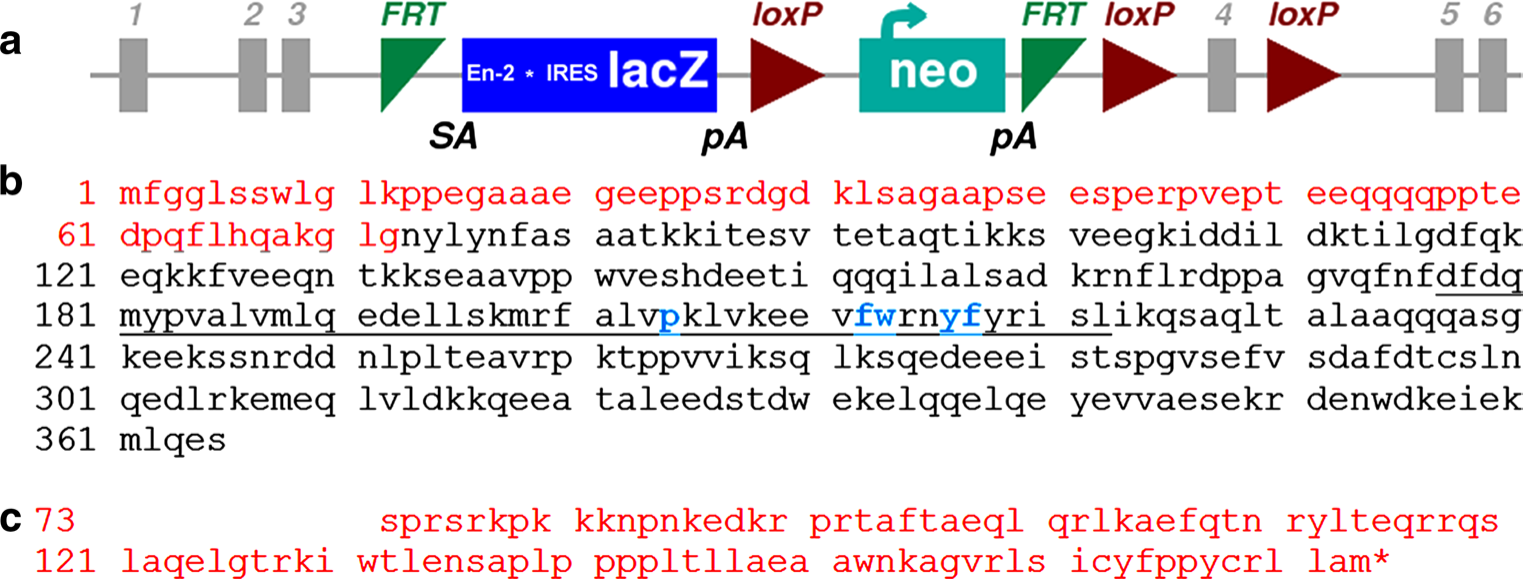
Schematic of the targeted mutation 1a allele (*Syap1* ^*tm1a(EUCOMM)Hmgu*^) of the mouse *Syap1* gene (**a**, *details in text*), amino acid sequence of mouse Syap1 protein (**b**), and fusion protein (*red amino acids*) expressed in knock-out mouse (**b**, **c**). Syap1 exons 1–3 encoding the *red* sequences in **b** are spliced to sequences encoding a fragment of mouse engrailed-2 protein shown in **c**. Conserved amino acids of the BSD domain (*underlined* in **b**) are marked in *blue*

After expansion of the clone according to the EUCOMM recommendations, 10–15 ES cells were injected into BALB/c host blastocysts. Injected embryos were cultured for 3–4 h to recover and then transferred into the right uterus horn of 2.5 dpc pseudopregnant CD1 surrogate mothers. The offspring were selected based on their chimeric coat color. High-percentage male chimeras were bred with C57BL/6N females, and the F1 offspring were genotyped by PCR for the neomycin transferase selection marker (Neo) and by Southern analysis. Due to the X-chromosomal location of *Syap1*, all mutants of the F1 generation were heterozygous females which were mated to C57BL/6J males. Mutants of the F2 generation were intercrossed in order to produce homozygous mutant females. Due to the *engrailed*-*2* splice acceptor site (SA) and a coding region of the mouse engrailed-2 protein (En-2) upstream of *lacZ,* a bi-cistronic transcript is generated producing a fusion protein consisting of a short Syap1 fragment translated from exon-1 to exon-3 of *Syap1* and an En-2 fragment (Fig. 1). The Syap1 fragment of this protein is predicted to be non-functional as it lacks the conserved BSD domain (Doerks et al. 2002) which has been shown to be a requirement for Syap1 function (Yao et al. 2013). Therefore, unless there is residual splicing from exon-3 to exon-4, mice homo- or hemizygous for the *Syap1*^*tm1a*^ allele will be *Syap1* null mutants. Due to an IRES sequence upstream of *lacZ*, *β*-galactosidase is translated from the bi-cistronic mRNA and can provide information of Syap1 expression in wild-type mice. The targeting construct *Syap1*^*tm1a*^ also allows for reconstitution of gene function and the generation of a *Syap1*^*tm1c*^ conditional knock-out allele following exposure to site-specific Flp recombination.

### RNA isolation and quantitative real-time PCR

RNA was isolated from adult mouse cortex with the TRI Reagent (Sigma-Aldrich) following the manufacturer`s instructions. Contamination by genomic DNA was reduced by a subsequent DNase treatment of the isolated RNA. 500 ng–1 µg of total RNA was used for cDNA synthesis employing random primers and SuperScriptIII Reverse Transcriptase (Invitrogen). The cDNA was diluted 1:5 in Tris-HCl with pH 8.0 containing 10 % bovine serum albumin (BSA) and used for quantitative real-time PCR with the Luminaris HiGreen qPCR Master Mix (Thermo Scientific) on a Lightcycler 1.5 (Roche) as previously described (Briese et al. 2016). Sequences of the primers are listed in Supplementary Table S1.

### Antibodies

The polyclonal Syap1 antiserum generated against affinity-purified human Syap1 was obtained from Proteintech, Chicago, IL, USA (rabbit, 16272-1-AP; Western Blot (WB): 1/3000, immunocytochemistry (IC): 1/200)). Antibodies against *α*-tubulin (T5168; WB: 1/4000, IC: 1/2000) and acetylated *α*-tubulin (mouse, clone 6-11b-1; T7451; WB: 1/4000, IC: 1/2000) were obtained from Sigma. Tyrosinated *α*-tubulin antibody (rat, clone YL1/2; ab6160; WB: 1/2000), histone H3 antibody (rabbit, ab8580; WB 1/30000), tyrosine hydroxylase (TH) antibody (chicken, ab76442; IC: 1/500), and GFP antibody (chicken, ab 13970; IC: 1/2000) were supplied by Abcam, Cambridge, UK. Antibodies against Akt (rabbit, #9272; WB: 1/4000), Akt (mouse, #2966; IC: 1/50), phospho-Akt Ser^473^ (rabbit, #4060; WB: 1/4000; IC: 1/200), and phospho-Akt Thr^308^ (rabbit, #9275; WB: 1/1000) were purchased from Cell Signaling, Danvers, MA, USA. Rabbit anti-GFP antibody (Sc-8334; WB: 1/4000) was obtained from Santa Cruz Biotechnology, Dallas, TX, USA, rabbit antiserum against Calnexin (ADI-SPA-860; WB: 1/4000) from Enzo Life Sciences, Farmingdale, NY, USA. Monoclonal antibody against GAPDH (CB1001; WB: 1/4000) was supplied by Calbiochem, Billerica, MA, USA, and antibodies against *γ*-adaptin (mouse, A36120 lot 1; IC: 1/1000) and anti-GM130 (mouse, 610822; IC: 1/500) were obtained from BD Biosciences, San Jose, CA, USA. Goat anti-choline acetyltransferase (AB144p; IC: 1/1000) and mouse anti-NeuN (MAB377; IC 1/400) were ordered from Millipore, Darmstadt, Germany, and mouse monoclonal antibody against CalbindinD-28K was purchased from Sigma, St. Louis, Missouri, USA (mouse, C9848; IC: 1/1000). Guinea pig anti-vGlut1 (135304; IC: 1/2000) and mouse anti-GFAP (173011; IC: 1/2000) were obtained from Synaptic Systems, Goettingen, Germany. Secondary antibodies, donkey anti-rabbit Cy3 (711-165-152), donkey anti-mouse Cy5 (715-175-150), donkey anti-chicken Alexa488 (703-545-155), donkey anti-goat Alexa488 (705-545-147), goat anti-rabbit peroxidase coupled (111-035-003; WB: 1/5000), and goat anti-mouse (115-035-003; WB: 1/5000) were provided by Jackson Laboratory, Farmington, CT, USA. Fluorophore-coupled secondary antibodies were from Jackson Laboratory (IC: 1/800), except goat anti-mouse IgG1-gamma chain Cy5-labeled antibody (IC: 1/500) was obtained from Abcam (ab136127). Horseradish peroxidase-coupled goat anti-rat antibody was obtained from Rockland, Limerick, PA, USA (612-103-120; WB: 1/5000); Phalloidin Fluor546 (IC: 1/30) was supplied by Life Technologies Corporation Grand Island, NY, USA; and *α*-Bungarotoxin Alexa647 conjugate was purchased from Thermo Fisher Scientific, Erlangen, Germany (B-35450; IC: 1/500).

### Immunohistochemistry

Adult wild-type or *Syap1* knock-out littermate mice (age between 6 and 8 weeks) were euthanized by the application of CO_2_ for approximately 3 min. Animals were first perfused *trans*-cardially with phosphate-buffered saline (PBS) containing 0.4 % heparin to remove the blood from the arteries and veins. To fix the tissues, perfusion was performed with 4 % paraformaldehyde in PBS (PFA). Brain and lumbar spinal cord were carefully dissected and post-fixed for at least 2 h with 4 % PFA at 4 °C followed by three washing steps with PBS and stored at 4 °C in PBS. The tissues were embedded in 6 % agarose, and 30- to 40-µm-thick vibratome sections were produced and stored in cryo-protection buffer (30 % ethylene glycol, 25 % glycerol, 0.05 M phosphate buffer pH 7.4) at −20 °C until use. Prior to immunolabeling, the sections were transferred to a 24-well dish and washed three times with PBS to remove cryo-protection buffer. Blocking and permeabilization was carried out in blocking buffer (0.3 % Triton X-100, 0.1 % Tween-20, 10 % donkey serum (Bio-Rad, USA, C06SBZ) in PBS) for 1 h at room temperature and 1 h at 37 °C. The sections were incubated in primary antibodies diluted in blocking buffer for 2 days at 4 °C on a shaker followed by three washes in washing buffer (0.3 % Tween-20, 0.1 % Triton X-100 in PBS) for 15 min each and incubated with secondary antibodies in blocking buffer for 2 h at room temperature. Three additional washing steps (45 min each) were performed prior to 4′,6-diamidine-2′-phenylindole dihydrochloride (DAPI, 0.4 µg/ml) staining. Sections were washed with PBS and embedded in FluorSaveTM (Millipore).

To investigate the distribution of Syap1 at neuromuscular synapses, the gastrocnemius muscles from adult Bl/6 WT and *Syap1* KO mice were dissected after perfusion. After 2 h post-fixation, single fibers were picked out and washed in 0.1 M glycine buffered with PBS. To visualize postsynaptic structures, nicotinic acetylcholine receptors were stained with *α*-bungarotoxin-Alexa-Fluor 647 in PBS for 20 min at RT and the fibers were washed for 20 min in PBS. Subsequently, the fibers were permeabilized and blocked with 10 % donkey serum, 0.3 % Triton X-100 for 1 h. The presynaptic site of the neuromuscular end plate was labeled with anti-synaptophysin. Antibodies were diluted in blocking solution and incubated over night at 4 °C. Subsequently, the tissue was washed three times in PBS for 10 min each, before the appropriate secondary antibodies were added. Finally, the fibers were washed again three times with PBS and mounted on a glass slide.

### Immunocytochemistry

For immunocytochemistry, cells cultured on 10-mm coverslips were washed once with pre-warmed PBS and subsequently fixed with 4 % PFA for 10 min at 37 °C. After fixation, the motoneurons were permeabilized with blocking buffer containing 0.1–0.3 % Triton X-100 and 10 % donkey serum in PBS for 30 min at room temperature. Primary antibodies were diluted in blocking buffer and incubated over night at 4 °C. Cells were then washed 5 times for 5 min each with washing buffer (0.1 % Triton X-100, 0.2 % Tween-20 in PBS) and incubated with secondary antibodies diluted in freshly prepared blocking buffer for 1 h at room temperature. Subsequently, the motoneurons were washed again twice for 5 min, followed by 10 min of DAPI (0.4 µg/ml) staining. After three additional washes, the coverslips were washed again once with ddH_2_O and mounted on glass slides using FluorSaveTM (Millipore).

### Lentivirus production for *Syap1* knockdown and controls

pLL3.7 lentiviruses were produced as previously described (Subramanian et al. 2012), expressing either shRNA against *Syap1* or a mismatch shRNA, both co-expressing a GFP reporter gene as internal infection control. The knock-down vector for *Syap1* and a mismatch shRNA (Selvaraj et al. 2012) were obtained by cloning a *Syap1* (5′-TGA GAC AAT TCA ACA GCA GAT TCA AGA GAT CTG CTG TTG AAT TGT CTC TTT TTTC-3′) and a mismatch shRNA sequence (5′-GAA AAA AGG TTA GAA ACT TAA AGT GTT CTC TTG AAA CAC TTT AAG TTT CTA ACC A-3′) into the pLL3.7 lentivirus. In general, infection levels above 90 % were achieved.

### Embryonic mouse motoneuron culture

Motoneurons were isolated and enriched from lumbar spinal cords from E13.5 mouse embryos as described (Wiese et al. 2010). The lumbar spinal cord was dissected and ensheathing meninges removed. The tissue was trypsinized (0.1 %) for 15 min at 37 °C and subsequently gently triturated until a uniform cell suspension was obtained. Motoneurons were enriched by p75-antibody panning [5 ng/ml to precoat Nunclon Δ surface culture dishes (Sigma); p75-antibody clone MLR2 was originally generated and provided by the R. Rush Lab, Flinders University, Adelaide, Australia; (Rogers et al. 2006)]. By this method, a motoneuron culture of 90 % purity is achieved (Wiese et al. 1999). Cells were plated on poly-DL-ornithine hydrobromide (PORN, Sigma P8638) and laminin-111 (Invitrogen, Thermo Fisher Scientific, Waltham, MA USA 23017-015)-coated 10-mm coverslips in CELLSTARtissue culture dishes (Greiner Bio-One GmbH, Frickenhausen, Germany) or on 24-well Nunclon Δ surface culture dishes, depending on the experiment. The motoneurons were grown in neurobasal medium, 500 µM GlutaMAX (Invitrogen), 2 % B27 supplement (Invitrogen), 2 % horse serum (Linaris, Dossenheim, Germany), and 5 ng/ml BDNF or 5 ng/ml CNTF. 50 % of the culture medium was first replaced at day 1 and then every second day. Appropriate lentiviral infections were performed in a small volume prior to the plating of the cells. Infected motoneurons were detected by GFP reporter expression from lentiviral constructs.

### Stimulation experiments

Motoneurons were cultured for 5 DIV in the presence of CNTF (5 ng/ml) and serum-starved overnight. The cells were then stimulated with 0.1 % BSA-coated BDNF (20 ng/ml) for the indicated time periods or treated with 0.1 % BSA (unstimulated control), washed once with PBS, and directly extracted in Laemmli buffer. Subsequently, equal volumes were analyzed by Western blot.

For immunocytochemical analysis of the pAkt^Ser473^/Akt ratio in stimulated cells, motoneurons were grown for 5 DIV, starved for 6–7 h in Neurobasal medium containing B27 (insulin free; Invitrogen), and pulsed for 5 min with BDNF (20 ng/ml). Unstimulated cells were cultured for 5 DIV in the presence of 5 ng/ml BDNF. Subsequently, the cells were immediately washed with PBS, fixed with 4 % PFA, and stained against pAkt^Ser473^, Akt, and GFP. The images were taken at an Olympus Fluo View FV1000 confocal microscope and quantified by using the average intensity signals of the *z*-stacks. Technical background values (no tissue) were subtracted from the mean intensity values.

### Western blot analysis

Western blots were performed following standard procedures. Motoneurons were directly lysed in Laemmli buffer. Tissues dissected from adult mice (killed by cervical dislocation) were lysed in RIPA buffer followed by Bradford protein assay. Laemmli buffer was added to the samples to adjust for a final concentration of 2× and boiled for 10 min at 99 °C. 20 µg of protein was loaded. Incubation of the blots with primary antibodies was performed overnight at 4 °C in a 5 % milk (for phosphoprotein detection of 5 % BSA) solution followed by at least three washing steps. Secondary antibodies were added for 1 h at room temperature; subsequently, the blots were washed at least three times and developed with either ECL or ECL Advance systems (GE healthcare. Munich, Germany). ImageJ was used for densitometric quantification of specific signals, and unspecific background was subtracted.

### Survival assay, axon length measurements

The survival of motoneurons was quantified by plating 1000 cells per well (four-well dish). On day one, the number of motoneurons in a marked area in the center of the well containing about 20–100 cells was counted. After 7 DIV, the counting was performed again and the survival rate was calculated. For axon length measurements, 1500–2000 cells per coverslip were cultured and fixed after 7 DIV and stained for *α*-tubulin. At least 50 cells per experiment were examined under double-blinded conditions regarding genotype and cell treatment.

### Microscopy

Imaging for axon length or mouse brain section overview was performed with a Keyence 0.43 microscope using a NIKON PlanAPO 20x or 2x objective, respectively. Axon length measurements were taken using ImageJ software (Rasband, W. S., ImageJ, US National Institutes of Health, Bethesda, Maryland, USA, http://imagej.nih.gov/ij/, 1997–2015). 12-bit images for Syap1 localization in cultured motoneurons and mouse brain sections were taken using an Olympus Fluoview FV1000 confocal microscope with a FVD10 SPD spectral detector and diode lasers of 405, 473, 559, and 635 nm. The image *x-*, *y-*, and *z*-stacks were acquired with an Olympus UPLSAPO 10× (air), 20× (air), or 60× objective (oil, numerical aperture: 1.35). For intensity measurements, average intensities were determined from 12-bit raw images and were corrected for technical background (no tissue). Structured illumination microscopy was performed at a Zeiss ELYRA S.1 microscope using a PLAN APOCHROMAT 63× objective (oil, numerical aperture 1.4) generating 16-bit images. Unless otherwise noted, motoneuron images show the maximum projection of *z*-stacks represented in RGB format (8-bit per color channel). Identical confocal imaging settings and adjustments of brightness and contrast were used to compare anti-Syap1 immunoreactivity (IR) in cells under uninfected, mock-infected and *sh*-*Syap1*-infected conditions or in wild-type and knock-out tissue. Final processing of all images was performed with ImageJ and Photoshop 7.0 (Adobe). Pixel intensity-based co-localization of antibody-derived fluorescent signals was performed with an unbiased algorithmic method in two-color single-plane confocal images using the JACoP v2.0 plugin in ImageJ (Bolte and Cordelieres 2006). The values given represent Pearson’s correlation coefficient after Costes’ randomization (Costes et al. 2004) (mean ± SEM) for pixels, where two channels, representing corresponding immunoreactivity, are above a calculated intensity threshold at which pixels do not show any statistical correlation.

### Statistical analysis

At least three independent experiments were performed for statistical analysis with the GraphPad Prism 4 software. Unless indicated otherwise, data are expressed as mean ± standard error of the mean (SEM). In general, the analysis of variance (ANOVA) with Bonferroni post hoc test (if data were normally distributed) or for nonparametric statistics Kruskal–Wallis test with Dunn’s multiple comparison was applied.

## Results

### Initial phenotypic characterization of the *Syap1* gene-trap mouse

**Fig. 2:**
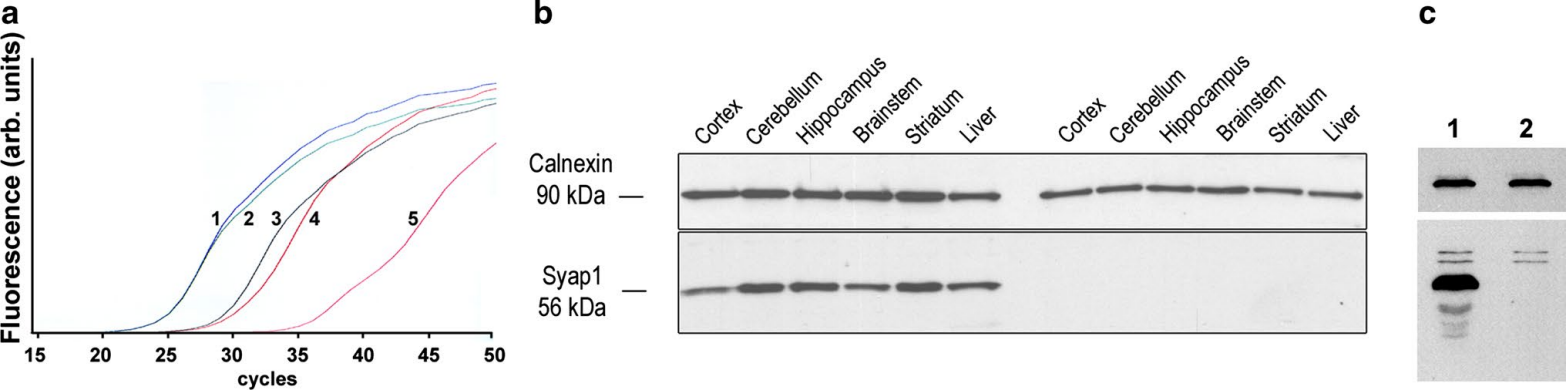
Verification of *Syap1* knockout and demonstration of Syap1 protein expression in different tissues. **a** qRT-PCR with mRNA from mouse cortex and primers connecting *Syap1* exon-3 and exon-4 demonstrates that intact transcript levels are reduced in the *Syap1* ^*tm1a*^ mutant (*curves* *3*, *4*) by a factor of ~30 compared to wild type (*curves* *1*, *2*). *Curve* *5* indicates background (no reverse transcriptase). **b** Lysates of the indicated tissues analyzed by Western blots show comparable levels of Syap1 protein expression in all tested brain regions of wild**-**type mice. However, the protein is also detected in non-neural tissues such as liver (*left half of blot*). No Syap1 signals are obtained for tissues from *Syap* ^*tm1a*^ mutant mice (*right half of blot*). **c** No trace of the Syap1 protein is detected by Western blots in lysates of hippocampus from *Syap* ^*tm1a*^ mutant (*lane 2*) even after increased protein loading and extended exposure. The two weak *upper bands* are unspecific, and the weak *lower bands* presumably represent Syap1 degradation products. *Top parts of blots* in **b** and **c**: Calnexin signals as loading controls

Heterozygous X-chromosomal *Syap1* gene-trap mice (*Syap1*^+*/tm1a*^) in C57BL/6N background were crossed to C57BL/6J mice in order to produce mutant males and females and to reduce non-C57BL/6J genetic background. Hemizygous *Syap1*^*tm1a*^ F2 males were crossed to *Syap1*^+*/tm1a*^ heterozygote females to obtain homozygous *Syap1*^*tm1a/tm1a*^ (*Syap1*^-/-^) female animals. Frequencies of offspring genotypes so far were compatible with an assumption of reduced viability of females homozygous for the *Syap1*^*tm1a*^ allele (data Table 1). No obvious differences in size or morphology between homozygous, hemizygous, heterozygous mutant, and wild-type littermates of the same sex were noted. The weights of mutant and wild-type males were not significantly different (weight ratio mutant/wild-type 0.996 ± 0.025, 4 age groups, 12 mutant and 12 wild-type animals, *P* = 0.56). Since the *tma1* insertion allele does not delete *Syap1* coding sequences (Fig. 1), we determined whether any residual intact *Syap1* transcript or protein was generated by exon-3 to exon-4 splicing (ignoring the closer *En*-*2* splice acceptor site (SA) of the *tm1a* insertion). qRT-PCR of cDNA isolated from wild-type and *Syap1*^-*/*-^ mutant brain with primers connecting exon-3 to exon-4 showed a reduction in the mutant of transcripts expressing these exons to 5.68 ± 0.54 % of wild-type levels (*n* = 6 independent experiments) (Fig. 2a). Transcripts containing exon-8 and exon-9 were reduced in the mutant to 14.8 ± 2.9 % (*n* = 3). Using an antiserum against Syap1 (characterized in Online Resource 1), we observed in Western blots of tissue lysates from wild type a clear Syap1 signal for all brain areas, for spinal cord, for sciatic nerve, but also for non-neuronal tissues such as diaphragm (Fig. S2a) and liver at the expected apparent relative molecular weight of 56 kDa (Fig. 2b). Corresponding tissue lysates from *Syap1*^-*/*-^ mutant mice showed no Syap1 signal. When more protein was loaded and exposure was extended, background signals and Syap1 degradation products showed up in the wild-type lane, but no Syap1 signal was detected in tissues from mutant mice (Fig. 2c). Under these conditions, the wild-type signal of hippocampal homogenate was still detectable when a 1:100 dilution of the lysates was loaded (Fig. S2b). These results demonstrate that Syap1 is abundant in nervous tissue, but it is not a nervous system-specific protein. In view of the observation that in the *Syap1* mutants intact Syap1 protein is absent (or reduced to less than 1 % of its normal level) and that in the Syap1–En2 fusion protein more than 80 % of the Syap1 primary structure is missing [including the functionally relevant BSD domain (Doerks et al. 2002; Yao et al. 2013)] (Fig. 1), we conclude that the *Syap1*^*tm1a*^ allele may be regarded as a null allele on a functional level.

**Table 1:** Genotype distributions of offspring (**F1**) of the indicated parental (**P**) crosses for the first three generations of *Syap1* ^*tm1a*^ mutant mice

P	Y/+ × +/−				Y/− × +/−			
F1	Y/+	Y/−	+/+	+/−	Y/+	Y/−	+/−	−/−
	16	23	28	19	9	3	15	4

Distributions of progeny of wild-type males and heterozygotes are compatible with Mendelian inheritance (*P* = 0.30), and progeny of mutant males and heterozygotes could point to a deleterious effect of the *Syap1* ^*tm1a*^ allele (*P* < 0.01, Chi–square test)

### Syap1 distribution in the adult mouse brain

**Fig. 3:**
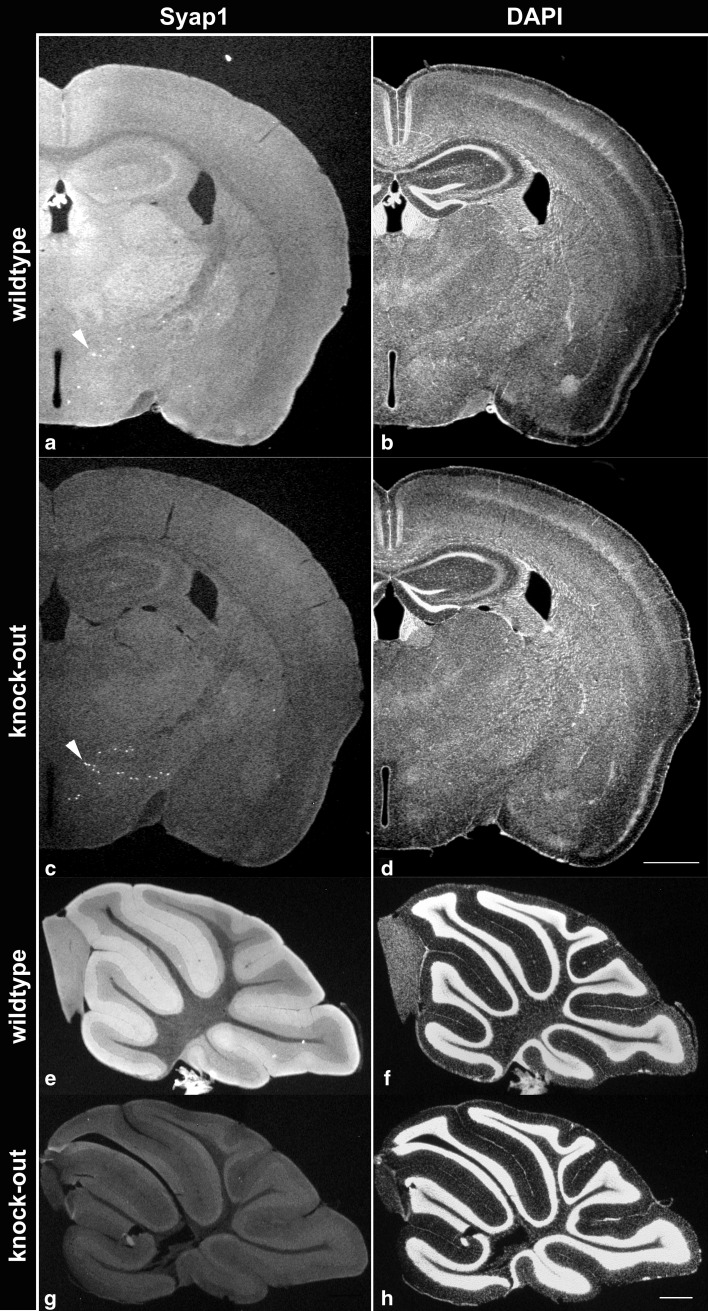
Sections of wild type (**a**, **b**, **e**, **f**) and *Syap1* knock-out (**c**, **d**, **g**, **h**) brains stained with Syap1 antiserum (*left*) and DAPI (*right*). In coronal sections (**a**, **c**), diffuse Syap1-specific immunolabeling is found throughout all parts of the brain, preferentially localized in the gray matter neuropil. *Arrowheads point* to hypothalamic cells that unspecifically cross-react with the Syap1 antiserum as they are detected both in wild-type and knock-out sections but not in controls without primary antibody (see Fig. S3). The *asterisk* indicates a thalamic region containing numerous cells with strong perinuclear Syap1-specific immunofluorescence labeling (enlarged in Fig. 5). Sagittal sections reveal particularly intense immunofluorescence for Syap1 in the molecular layer of the cerebellar cortex (**e**, **g**). *Scale bars* 1 mm (*upper*), 200 µm (*lower*) *panels*

With the availability of *Syap1* knock-out animals, it now is possible to reliably determine the distribution of Syap1 in wild-type tissues at the cellular and subcellular level. Since so far we have no information about the function of Syap1 in mammalian nervous system, we here give an overview on Syap1 localization in various parts of the mouse brain and report in more detail the distribution in selected regions. Comparison of coronal sections of wild-type and *Syap1* knock-out brains immuno-reacted with the Syap1 antiserum reveals intense Syap1-specific immunofluorescence throughout the brain with regionally distinct distribution patterns (Fig. 3). Fluorescent signals observed in both wild-type and knock-out sections (e.g., individual cells in the hypothalamus, arrowheads in Fig. 3a, c; distinct fiber-like structures in other brain areas, see below) are lacking in negative controls incubated without Syap1 antiserum (Fig. S3). This indicates the cross-reactivity of the antiserum with an unknown antigen and emphasizes the necessity of comparing wild-type and knock-out sections processed in parallel for specifically characterizing Syap1 distribution in the various brain areas, as done in the present study.

**Fig. 4:**
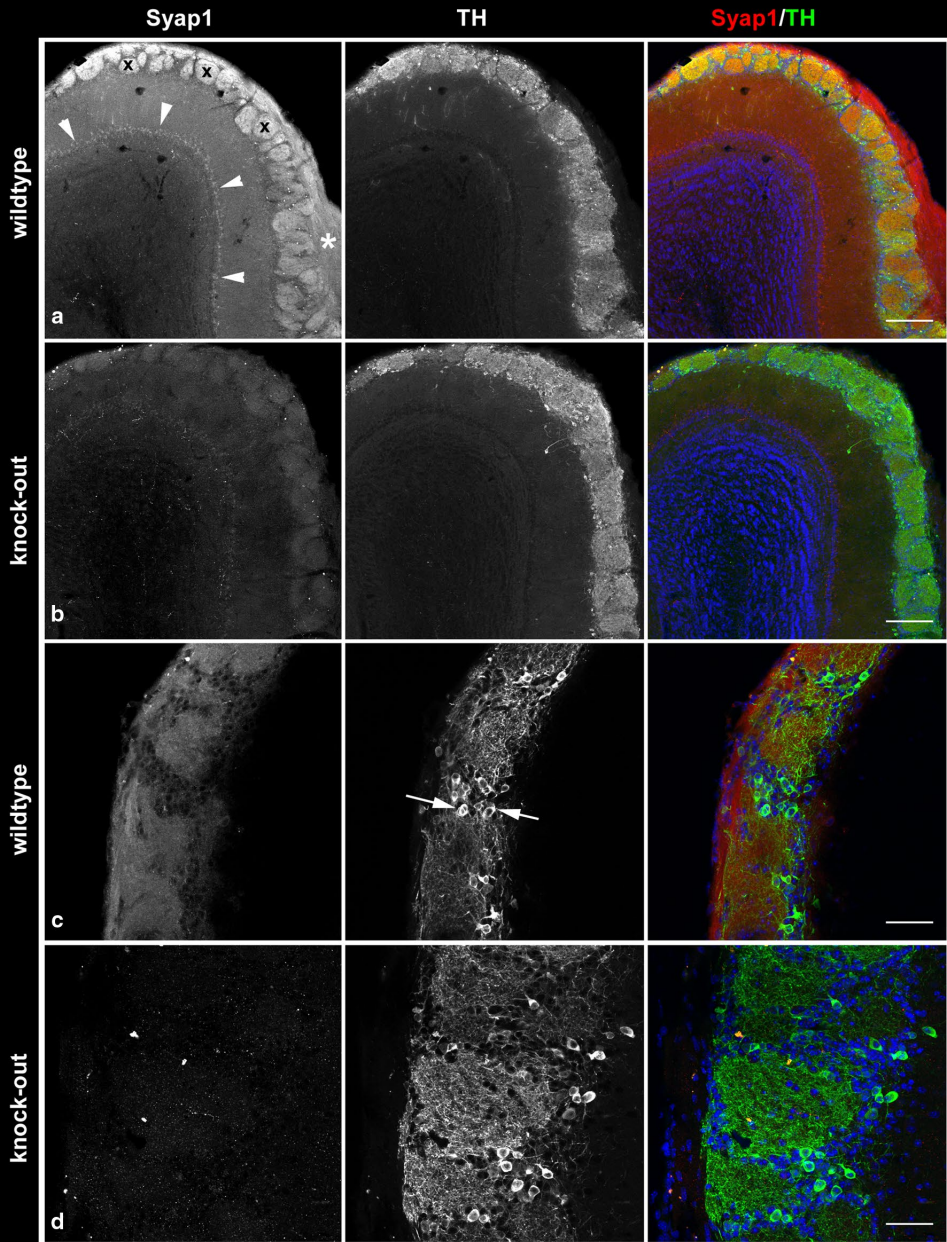
In the main olfactory bulb, intense Syap1-specific immunoreactivity is observed in olfactory nerve layer (*asterisk*) and glomeruli (*x*) of wild-type sections (**a**, **c**). Less intense Syap1 signals are found in the mitral layer (*arrowheads* in **a**). The Syap1 antiserum weakly cross-reacts with an unknown antigen in knock-out sections (**b**, **d**). Dual immunolabeling with anti-Syap1 antiserum (*left*) and anti-TH (*middle*) reveals Syap1 accumulation in glomerular neuropil. Nuclei are stained by DAPI [*blue in the overlay* (*right*)]. *Scale bars* 200 µm (*upper half*), 50 µm (*lower half*)

At low magnification, most brain areas display diffuse Syap1-specific immunolabeling (Fig. 3a, e), and comparison with neuronal localization patterns visualized by DAPI nuclear counterstaining (Fig. 3b, f) indicates a preferential localization of the protein in gray matter neuropil. This is obvious in cortical regions and hippocampus, and is especially prominent in the cerebellum, where the molecular layer displays particularly intense diffuse fluorescence while the granular layer and the cerebellar medulla are significantly less strongly labeled (Fig. 3e, f, see below). In the olfactory bulb, Syap1 immunolabeling is strongest in the olfactory nerve layer and olfactory glomeruli (Fig. 4a, left column), which contain the glutamatergic terminals of the olfactory nerve axons and are surrounded and delineated by tyrosine hydroxylase (TH) immunoreactive dopaminergic periglomerular cells and their processes extending into the glomeruli (Fig. 4a, middle column). While there is light Syap1 immunoreactivity in the mitral layer (arrowheads in Fig. 4a), there is no conspicuous staining observed in the perikarya of dopaminergic periglomerular cells (arrows in Fig. 4c).

**Fig. 5:**
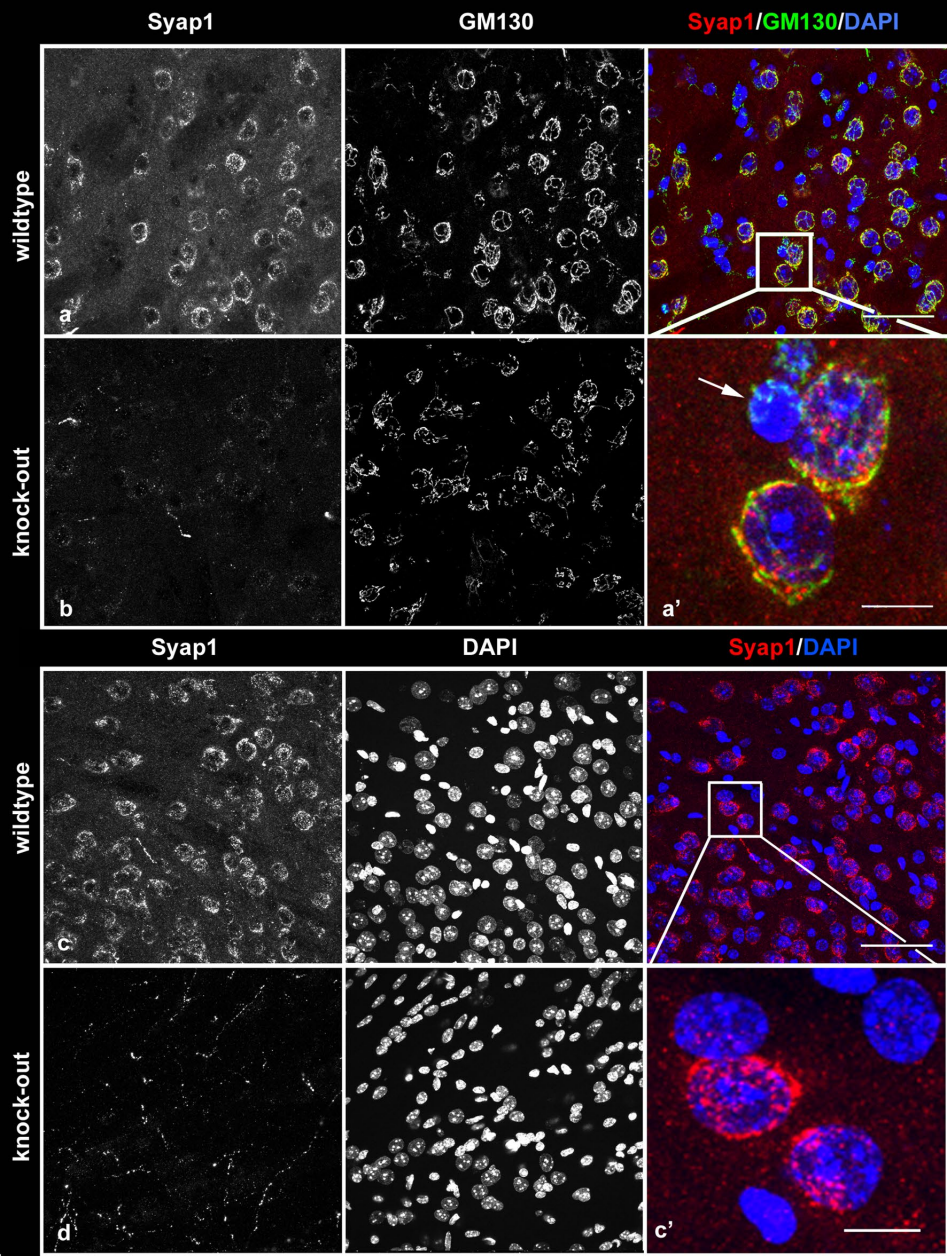
Syap1 distribution in the thalamus. **a** Enhanced staining of anti-Syap1 (*left*) in a certain region of the wild-type thalamus (indicated in Fig. 3a by an *asterisk*) is by double staining with the Golgi marker GM130 (*middle*) identified as perinuclear accumulation of Syap1-specific immunofluorescence near the Golgi apparatus. *Yellow color* in the enlargement in **a′** indicates overlap of Syap1 and GM130, and *arrow* points to GM130-immunoreactive Golgi apparatus in a presumably non-neuronal cell (as indicated by a densely DAPI-stained nucleus) lacking Syap1 immunolabeling. **c** This perinuclear accumulation of Syap1-specific immunofluorescence is confined to a subpopulation also of neurons as revealed in the enlargement (**c′**) by nuclei labeled with DAPI (*blue*) devoid of Syap1-specific immunofluorescence (*red*). Comparison with knock-out sections in **b** and **d** (*left and middle panels*) shows fine fluorescent fiber-like structures labeled due to unspecific cross-reaction of the anti-Syap1 antiserum. *Scale bars* 25 µm (**a**, **c**); 5 µm (**a′**, **c′**)

**Fig. 6:**
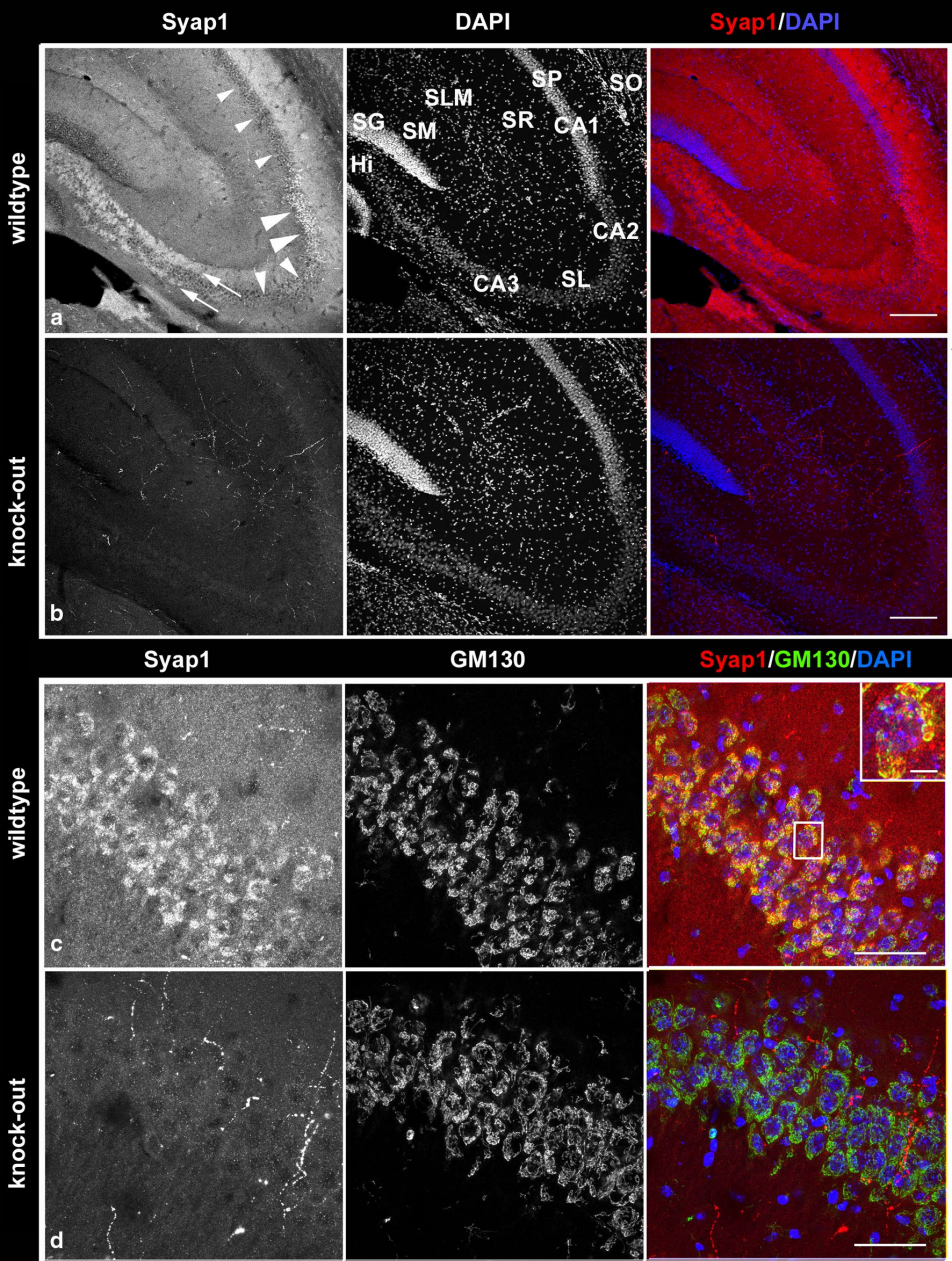
Syap1 distribution in the hippocampus. **a** In wild-type sections, Syap1-specific immunofluorescence is present in general neuropil, high levels of Syap1-labeling are observed in CA2 perikarya (*large arrowheads*), and Syap1 immunoreactivity is also detectable to a weaker extent in CA1 or CA3 perikarya (*small arrowheads*), compared with knock-out sections (**b**, **d**). The infra- and suprapyramidal mossy fiber pathways arising from dentate gyrus granule cells show intense Syap1-specific immunoreactivity (*arrows*). Syap1 is also found in the stratum lucidum. (**c**, **d**) The enlargement of the CA2 region double stained with anti-Syap1 and GM130 demonstrates the association of perinuclear Syap1 with the Golgi apparatus of these cells. Hi, hilus; SG, stratum granulosum; SL, stratum lucidum; SLM, stratum lacunosum-moleculare; SM, stratum moleculare; SO, stratum oriens; SP, stratum pyramidale; SR, stratum radiatum. *Scale bars* 200 µm (**a**, **b**), 50 µm (**c**, **d**), 10 µm (*inset* in **c**)

In the thalamus, a region of enhanced Syap1 immunolabeling is observed (marked by an asterisk in Fig. 3a, compared with knockout in b). At higher magnification (Fig. 5a, c), it becomes apparent that this is caused by perinuclear accumulations of Syap1-immunoreactive material in numerous neurons (identified by their typical nuclei in DAPI staining, Fig. 5a′, c′, or by double staining with anti-NeuN antibodies, a neuron-specific marker (Fig. S4b), in close association with immunofluorescence for GM130, a marker for the *cis/medial*-Golgi apparatus (Nakamura et al. 1995) in dual immunolabelings (Fig. 5a, a′). In astrocytes and presumably other non-neuronal cells displaying comparatively small and heterochromatin-rich nuclei in DAPI stainings, accumulation of Syap1-immunoreactivity near the Golgi apparatus is lacking (arrow in Figs. 5a′, S4a, b). Also, the fact that apparently not all neuronal nuclei are surrounded by Syap1-immunoreactivity (Fig. 5c′) indicates that Syap1 expression is under cell-(sub)type-specific control. This becomes even more obvious in the hippocampus. Here, we find low levels of Syap1-specific immunofluorescence in the pyramidal layer of CA1 and CA3 (small arrowheads in Fig. 6a), but high concentrations in practically all perikarya of the CA2 pyramidal layer (large arrowheads in Fig. 6a, at higher magnification in c). Again, association with GM130-immunoreactivity is notable in CA2 neuronal perikarya (Fig. 6c, inset). This observation induced us to ask whether the structure of the Golgi apparatus was noticeably altered when Syap1 protein was missing. We therefore compared GM130 staining in wild-type and Syap1 knock-out brain sections at higher magnification. No qualitative effect of Syap1 knockout on Golgi structure as revealed by light microscopical GM130 staining was noted (Fig. S6). Medium intense, diffuse Syap1-immunoreactivity is observed in the dentate gyrus stratum moleculare and in the CA stratum lacunosum-moleculare. In CA1, the strata oriens and radiatum display medium high and low immunoreactivity levels, respectively. While dentate gyrus granule cells display scarce perinuclear labeling, the hilus contains strong punctate Syap1-immunoreactivity (Figs. 6a, S5a) which extends into the CA3 region, forming two bands of particularly intense fluorescence adjacent to CA3 somata (arrows in Figs. 6a, S5b) corresponding to the supra- and infrapyramidal mossy fiber pathways, the suprapyramidal band extending throughout the stratum lucidum of CA3 into CA2 (Fig. 6a). In dual immunolabelings, partial co-localization of Syap1 with the vesicular glutamate transporter vGlut1 is observed in the mossy fiber pathway (Fig. S5a, b), indicating the presence of the protein in the particularly large glutamatergic mossy fiber boutons but also in additional non-vGlut1 immunoreactive elements.

**Fig. 7:**
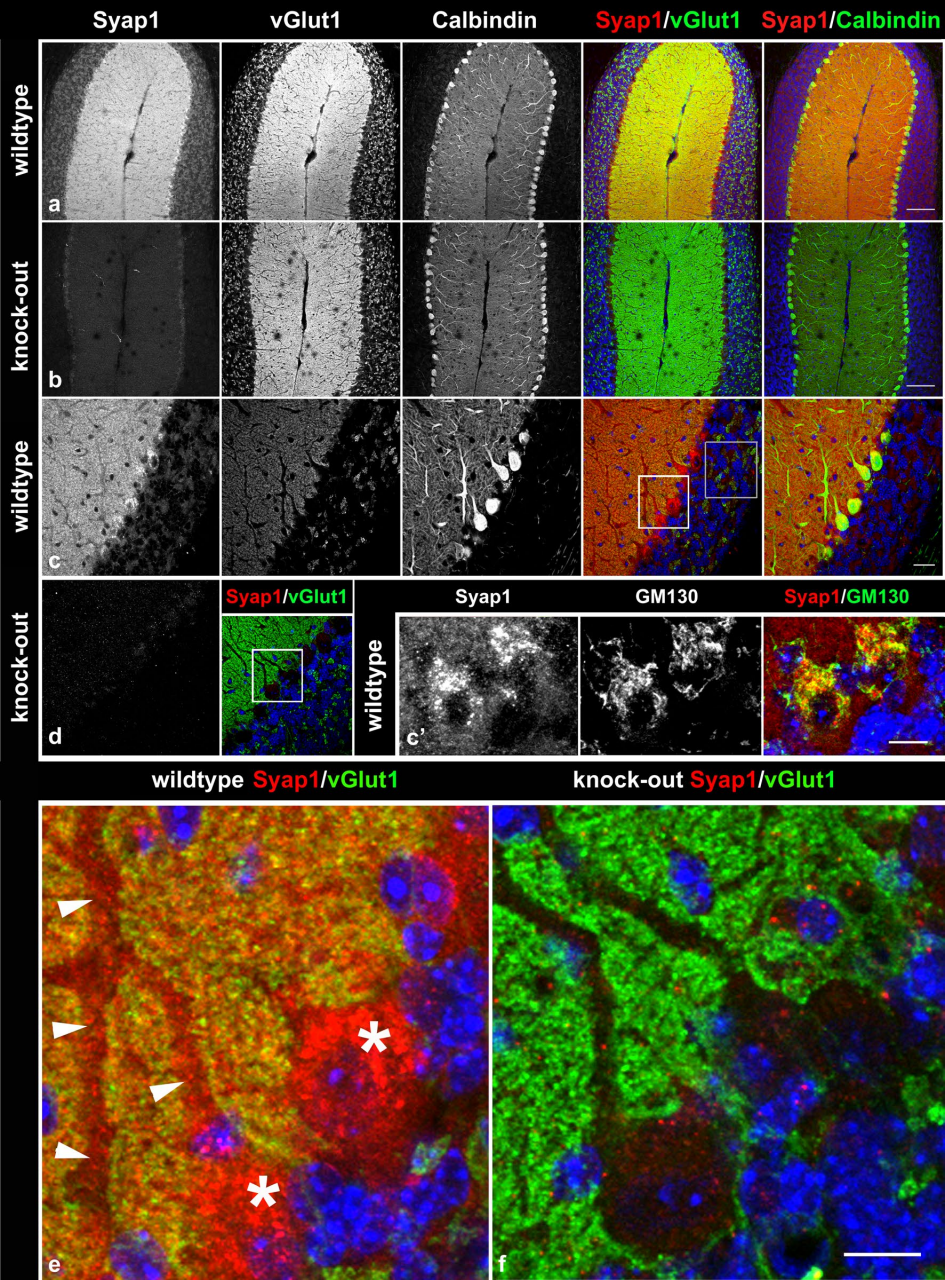
Syap1 distribution in the cerebellum. Syap1-specific immunofluorescence accumulations largely overlap with glutamatergic synaptic neuropil labeled by anti-vGlut1 staining which marks the vesicular glutamate transporter of glutamatergic synapses [compare wild-type (**a**, **c**, **e**) with knock-out sections (**b**, **d**, **f**)]. In enlargements (**e**, **f**) of the regions indicated by the white squares in **c** and **d,** the perinuclear accumulation of Syap1-specific immunofluorescence in Purkinje cells identified by calbindin double labeling (*center column* in **a**–**c**) is apparent (*asterisks* in **e**), but Syap1 is also detected in the large dendrites of these cells extending into the molecular layer (*arrowheads* in **e**). Again, Syap1 immunofluorescence partially overlaps with signals for the Golgi marker GM130 (*yellow* in **c′**, *rightmost image*). In the granular layer (*gray square* in **c**), Syap1-specific immunofluorescence overlaps with glutamatergic synapses but is also present in the perinuclear region of granule neurons and the local neuropil (enlarged in Fig. S5c). *Blue* nuclear DAPI. *Scale bars* 100 µm (**a**, **b**), 25 µm (**c**, **d**), 10 µm (**c′**, **e**, **f**)

**Fig. 8:**
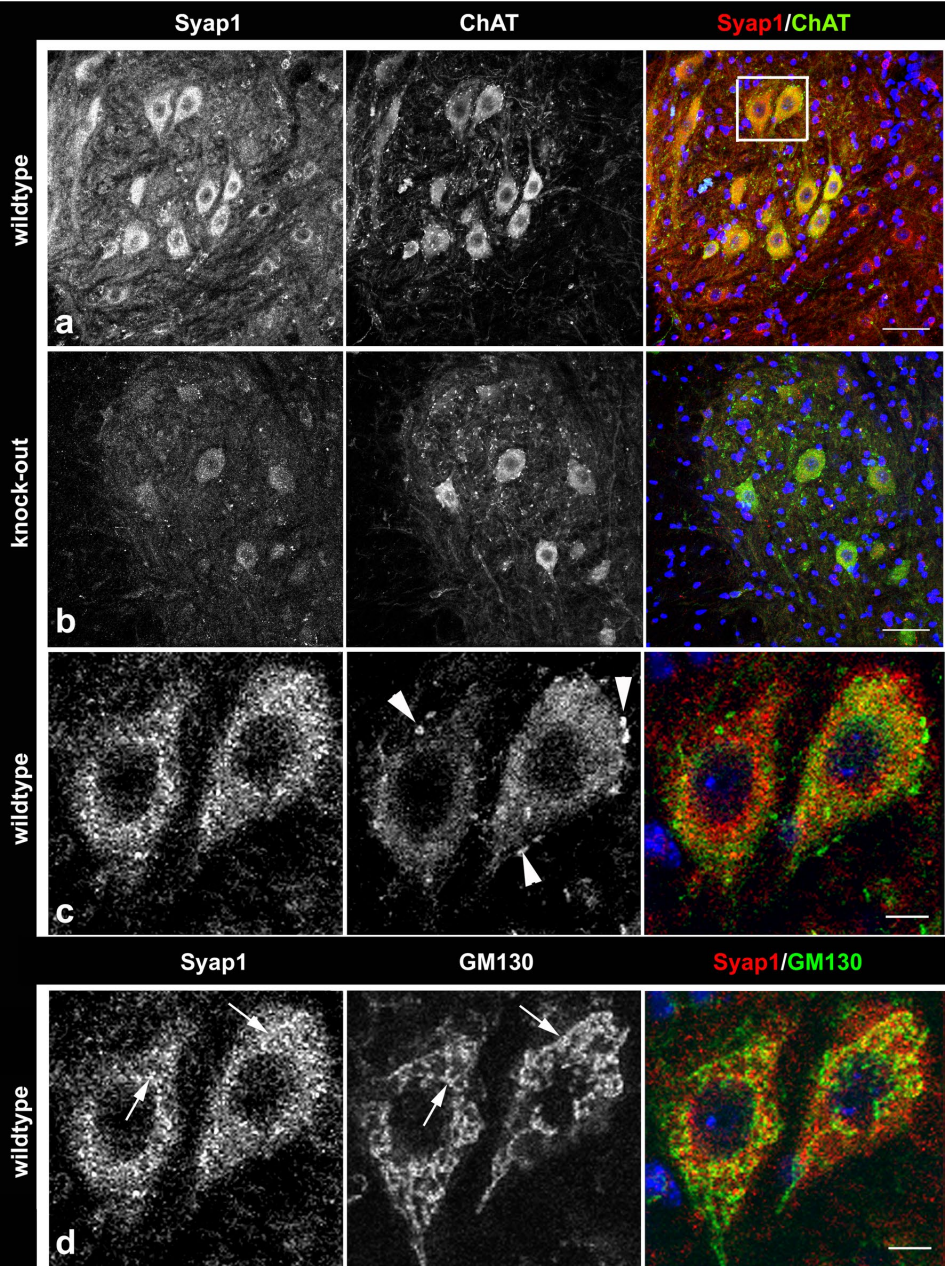
Syap1 (**a**, **c**, **d**,* left column*) in spinal cord neuropil and cholinergic cells (motoneurons) labeled by anti-ChAT antiserum (**a**–**c**, *middle column*). Compare wild-type section (**a**) with knock-out section (**b**). *Cells in white box* in **a** are shown enlarged in **c** and **d**. Cholinergic synaptic boutons (*arrowheads* in **c**) display little or no Syap1-specific immunofluorescence. Again, there is considerable overlap of Syap1 signals and GM130 staining (*arrows* in **d**). *Blue* nuclear DAPI. *Scale bars* 100 µm (**a**, **b**); 50 µm (**c**, **d**)

**Fig. 9:**
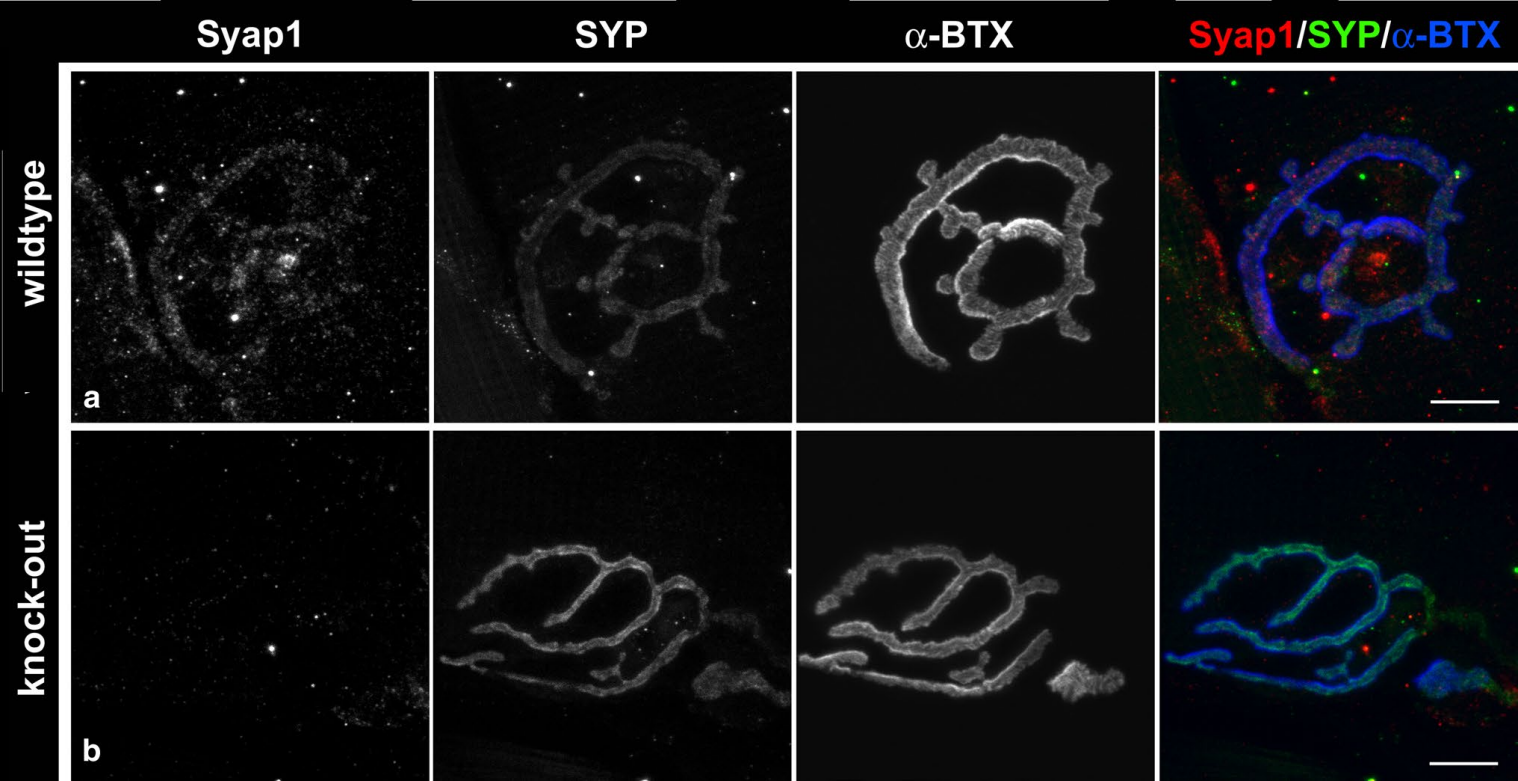
Syap1 at neuromuscular junctions. By comparison with the distribution of synaptophysin (SYP) and *α*-bungarotoxin (*α*-BTX), low levels of Syap1-specific immunofluorescence are identified at neuromuscular junctions, but Syap1 signals are also observed extra-synaptically in the muscle. Compare wild-type (**a**) with knock-out preparation (**b**). *Scale bars* 10 µm

In the cerebellum, pronounced Syap1 staining is observed in the perinuclear cytoplasm of Purkinje cells (Fig. 7a, c, e, asterisks), particularly concentrated close to the emerging dendritic tree, again associated with the Golgi complex identified by GM130 co-labeling (Fig. 7c′). In addition, Syap1 immunoreactive puncta are distributed throughout the Purkinje cell dendrites (arrowheads in Fig. 7e) and in the molecular layer neuropil between the larger dendrites where numerous glutamatergic synapses are formed by parallel and climbing fibers. Co-staining for vGlut1 reveals partial overlap of the immunoreaction signals (Fig. 7a, c, e, compares with knockout in b, d, f). In the granular layer, medium levels of Syap1 immunoreactivity are observed, again partially overlapping with vGlut1 immunoreactivity identifying the large mossy fiber boutons of cerebellar glomeruli, but with signals also in non-vGlut1-reactive puncta (Fig. 7c, gray square enlarged in Fig. S5c). In the spinal cord, some overlap of Syap1-specific staining with choline acetyl transferase (ChAT) immunoreactivity in motoneuron somata is seen (Fig. 8a, c compares with knockout in b), but cholinergic synaptic boutons (arrowheads in c) contain little or no Syap1. Again, conspicuous association of the Syap1 signal with GM130 immunoreactivity in motoneuron cell bodies is obvious (arrows in Fig. 8d). These latter findings induced us to investigate the distribution and possible functional aspects of Syap1 in cultured motoneurons. However, the cholinergic synapses at neuromuscular junctions and surrounding muscle tissue display only low levels of Syap1-specific immunofluorescence (Fig. 9).

### Subcellular localization of Syap1 in isolated motoneurons

**Fig. 10:**
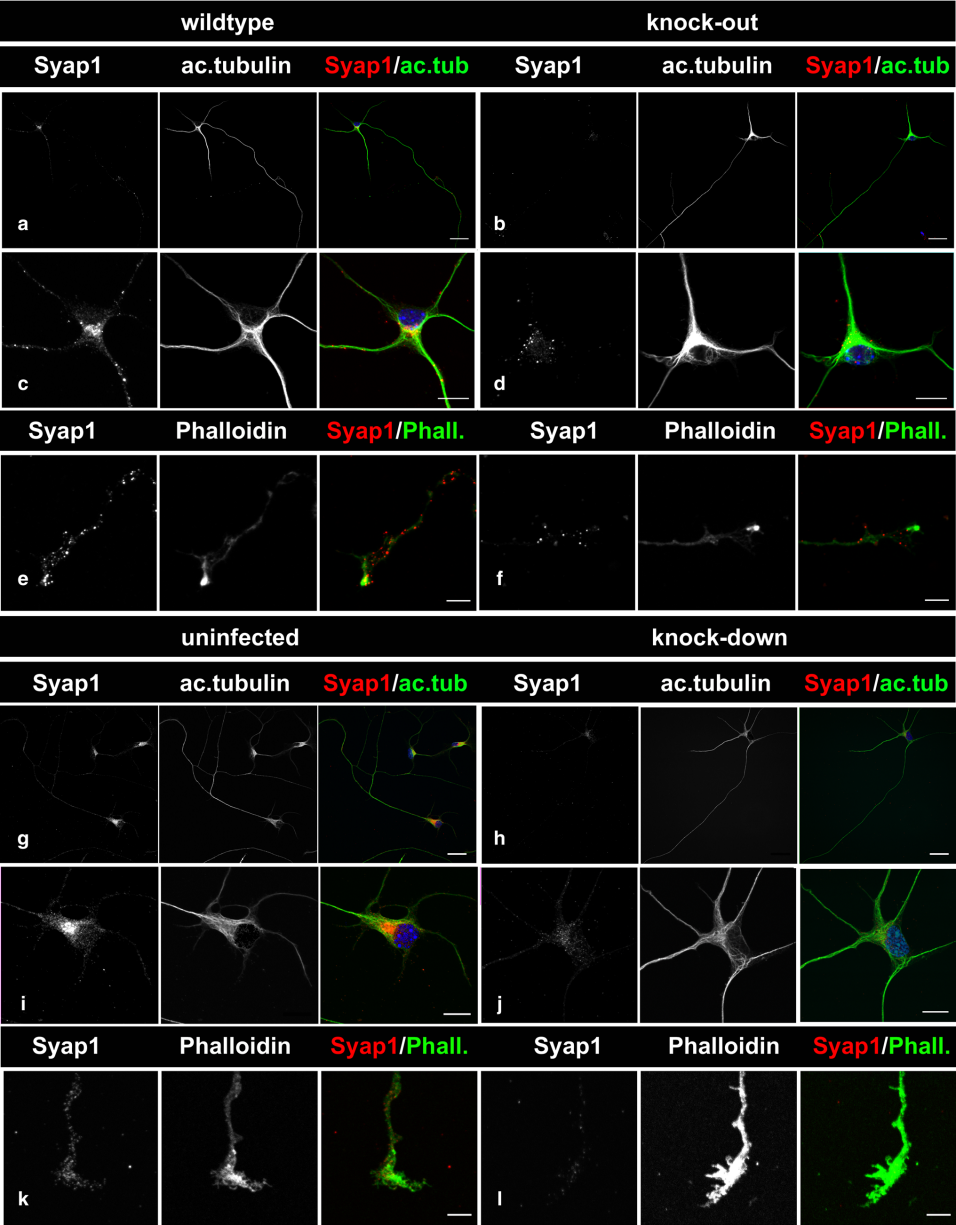
Localization of Syap1 protein in cultured primary motoneurons. **a** Representative images of primary motoneurons cultured for 5 days and stained against Syap1 and acetylated tubulin or co-stained with phalloidin, as indicated. *Syap1* knock-out cells (**b**, **d**, **f**) and shRNA–*Syap1*-treated cells (**h**, **j**, **l**) show a robust reduction in Syap1 immunoreactivity compared to wild-type (**a**, **c**, **e**) or uninfected (**g**, **i**, **k**) and sh-mismatch RNA (“mock”)-infected (not shown) controls. Syap1 signals are observed in the dendrites, soma, axon, and distal part of the axon. The magnified *images* of the somata (**c**, **i**) reveal punctate Syap1 staining in the cytoplasm and dendrites with a prominent perinuclear accumulation. Syap1 signals are also present in growth cones (**e**, **k**). *Blue* nuclear DAPI staining. *Scale bars* **a**, **b**, **g**, **h**: 25 µm; **c**–**f**, **i**–**l**: 10 µm

Motoneurons were isolated and enriched from E13.5 mouse embryos as described in “Methods” section. Cells from wild-type and knock-out embryos were cultured for 5 days, PFA-fixed and stained against Syap1 and acetylated tubulin, and counterstained with DAPI. In addition, wild-type cells infected either with the *Syap1* knock-down virus or with the mock virus were compared. Strong signals obtained by confocal immunofluorescence microscopy were seen with the Syap1 antiserum in wild-type cells (Fig. 10a, c, e, g, i, k), but no specific staining was obtained in *Syap1*^-*/*-^ knock-out cells (Fig. 10b, d, f). Residual fluorescence represents cross-reactivity of the primary antiserum as determined by omission of first antibody (not shown). A robust depletion of Syap1 signal after lentiviral *Syap1* knockdown was likewise apparent (Fig. 10h, j, l) by comparison with mock-treated (not shown) or uninfected control cells (Fig. 10g, i, k). Syap1 immunofluorescence revealed a punctate staining pattern which could be observed in somata, dendrites, axons, and growth cones of the motoneurons and was strongly reduced in knock-out or knock-down cells. The nucleus showed no distinct Syap1 signal. At higher magnification of the cell bodies, a strong perinuclear Syap1 signal was detected (Fig. 10c, i) which was clearly lacking in knock-out and knock-down neurons (Fig. 10d, j). In axonal growth cones, punctate Syap1 signals appeared densest in regions containing high levels of F-actin as identified by Phalloidin staining (Cooper 1987; Fig. 10e, k). Low levels of fluorescent signals throughout terminal axons in knock-out and knock-down neurons (Fig. 10f, l) confirmed the specificity of strong Syap1 signals in actin-rich growth cone regions.

### Syap1 localizes in close proximity to the Golgi apparatus and the *trans*-Golgi network

**Fig. 11:**
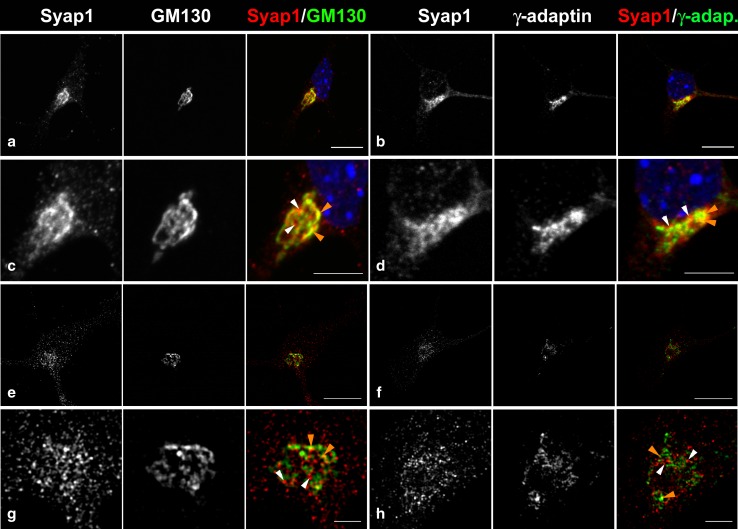
Syap1 accumulates close to the Golgi organelle. (**a**–**d**) Perinuclear Syap1 immunoreactivity partially co-stains with the *cis/medial*-Golgi marker GM130 (**a**, **c**) and with *γ*-adaptin, a component of the adapter protein complex 1 (**b**, **d**) (maximum intensity projections). *Blue* nuclear DAPI staining. (**e**–**h**) Super-resolution imaging by SIM of Syap1 and GM130 or *γ*-adaptin. Typical single optical sections are shown. (**e**, **g**) Syap1 and GM130 distribution, (**f**, **h**) Syap1 and *γ*-adaptin distribution. *Orange arrowheads* point to co-occurrence of both labels, and *white arrowheads* indicate Syap1-positive, GM130- or *γ*-adaptin-negative signals. *Scale bars* **a**, **b**, **e**, **f** 10 µm; **c**, **d** 5 µm; **g**, **h** 2.5 µm

In order to further define the subcellular localization of Syap1 in the cultured motoneurons, co-staining with antibodies against the *cis/medial*-Golgi apparatus marker GM130, and against *γ*-adaptin, a component of the adaptor protein complex 1 (AP1) particularly accumulated in the *trans*-Golgi network (Boehm and Bonifacino 2001), was performed (Fig. 11). Syap1 staining was found concentrated next to the nucleus in close proximity to the *cis/medial*-Golgi label (Fig. 11c, maximum intensity projections; orange arrowheads point at co-occurrence of both signals, while white arrowheads mark Syap1-positive, GM130-negative spots). Analyses of confocal optical sections revealed an average Pearson’s correlation coefficient after Costes’ randomization (PCC) of 0.66 ± 0.01 (pooled data of *N* = 50 cells, 4 independent cultures), indicating a non-random correlation between both immunoreactive signals. Syap1 and *γ*-adaptin IR (Fig. 11d) showed a PCC of 0.72 ± 0.01 (pooled data of *N* = 35 cells, 5 independent cultures). Confocal microscopy thus indicates a pronounced localization of Syap1 to the perinuclear Golgi apparatus, confirming observations in the brain sections. To examine the localization of Syap1 at even higher resolution, structured illumination microscopy (SIM) was performed. SIM is a super-resolution imaging approach using standard fluorescent probes as immunocytochemical tags and offers here an *x*–*y*-resolution in the range of 120 nm (see Schermelleh et al. 2010). SIM microscopy again showed a fine-structured, punctate distribution of Syap1 with a strong accumulation in the perinuclear Golgi region and a punctate staining throughout the cytoplasm. SIM optical sections reveal a close proximity to GM130 in the perinuclear area, but only a rare co-occurrence of both labels. Syap1 immunoreactivity did not follow the typical cisternae-like label of the *cis/medial*-Golgi marker GM130 (Fig. 11e, g; orange arrowheads point to co-occurrence of both signals, while white arrowheads point to Syap1-positive, GM130-negative labels). Similarly, SIM images reveal an even less frequent overlap of Syap1- and *γ*-adaptin IR (Fig. 11f, h; yellow arrowheads point to co-occurrence of both signals, while white arrowheads point to Syap1-positive, *γ*-adaptin-negative labels).

### *Syap1* knockout does not affect survival or axonal elongation in cultured primary motoneurons

**Fig. 12:**
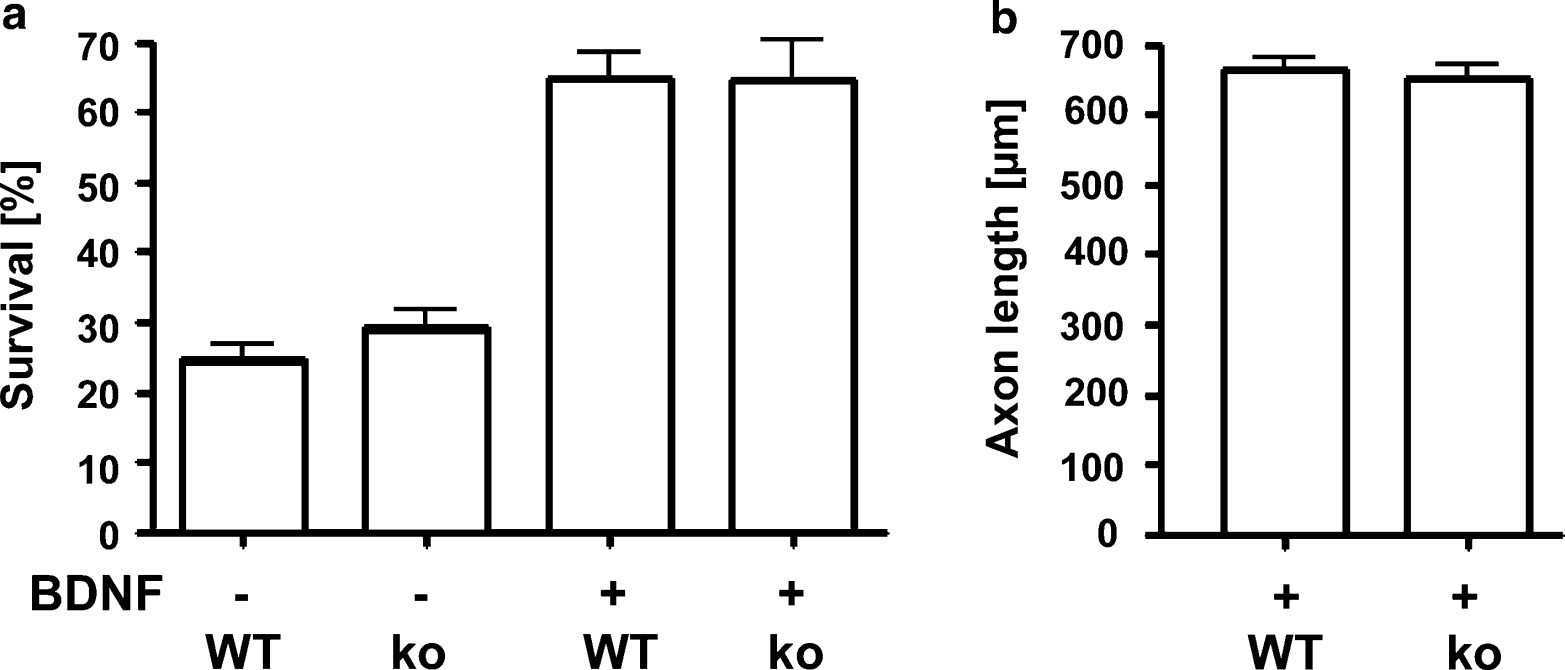
Knockout of *Syap1* does not modify survival or axonal outgrowth of primary motoneurons. **a** Quantification of the survival of primary motoneurons cultured for 7 DIV in the absence or presence of BDNF. Cells were counted on day 1 and again on day 7. There is no significant difference in the survival rate of motoneurons from *Syap1* knockout (ko) compared to wild-type (WT) embryos [*n* = 17 (WT) and 7 (ko) independent cultures]. Wild-type cells cultured without BDNF show highly reduced cell survival. **b** Axon length of primary motoneurons cultured for 7 DIV is not affected by *Syap1* knockout (*n* = 288 for WT and 237 for ko)

Primary motoneurons in culture require neurotrophic factors for survival and growth of axons and dendrites. Thus, the role of cell-autonomous factors and their effects on signaling pathways controlling axon elongation and growth cone differentiation can be analyzed using this system (Calof and Reichardt 1984; Forscher and Smith 1988; Arakawa et al. 1990; Sanes and Lichtman 1999; Sendtner et al. 2000). To study the function of Syap1, we therefore investigated the effect of *Syap1* knockout on survival and axon growth of cultured embryonic motoneurons (Fig. 12). After 7 days in vitro (DIV), there was no difference in the survival rate of *Syap1* knockout in comparison with wild-type motoneurons (*P* > 0.3), whereas a significant reduction in viability was observed when wild-type or knock-out neurons were cultured without the neurotrophic factor BDNF (*P* < 0.05) (Fig. 12a; without BDNF: wild-type: 24.9 ± 1.9 % (17); *Syap1* knockout: 29.4 ± 2.4 (7); with BDNF: wild-type: 64.6 ± 3.6 % (17); *Syap1* knockout: 64.5 ± 5.6 % (7); (in parentheses: numbers of independent cultures). The mean values for axon length (Fig. 12b) also did not differ significantly between the genotypes (axon length wild type: 660.5 ± 16.8 µm; *Syap1* knockout: 659.4 ± 19.3 µm; (*n* = 4 independent cultures; numbers of cells pooled per condition: wild type: *N* = 288, *Syap1* knockout: *N* = 237). These results indicate that Syap1 does not play a major role in microtubule dynamics or other molecular mechanisms relevant for neurite outgrowth (Paglini et al. 1998; Sendtner et al. 2000; Rossoll et al. 2003; Jablonka et al. 2007; Selvaraj et al. 2012).

### Syap1 deficiency apparently does not affect total Akt phosphorylation in primary motoneurons

**Fig. 13:**
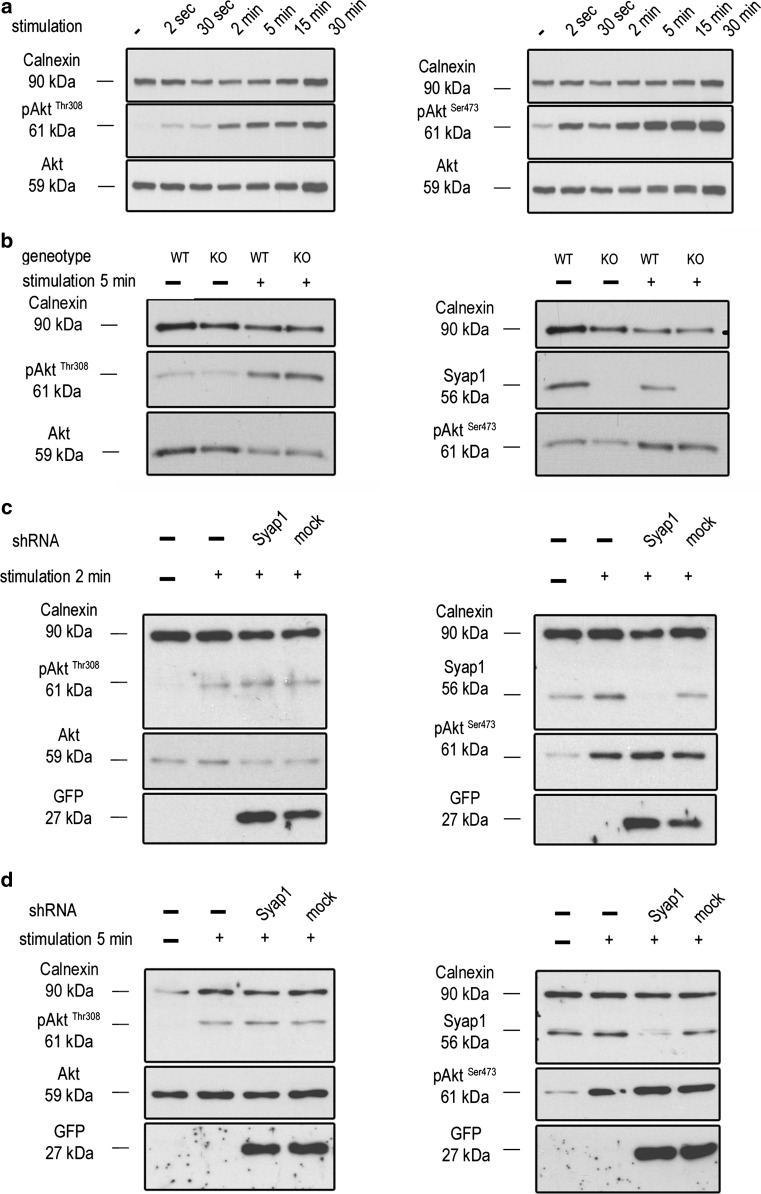
*Syap1* knockdown or knockout does not significantly influence total Akt phosphorylation at Thr^308^ and Ser^473^ in primary motoneurons. **a** Western blot of serum-starved cells stimulated with BDNF (20 ng/ml) in a time series ranging from 2 s to 30 min. Maximum Akt Thr^308^ (*left blot*) and Ser^473^ (*right blot*) phosphorylation is achieved after 2–5 min of neurotrophin stimulation. **b** Western blots of motoneurons from wild-type and *Syap1* knock-out embryos stimulated for 5 min with BDNF did not reveal a reduction in Akt phosphorylation at Thr^308^ (*left blot*) or Ser^473^ (*right blot*) due to *Syap1* knockout. Calnexin and pan-Akt served as loading controls while GFP levels indicate a positive infection of the cells. **c**, **d** *Blots* of *Syap1* shRNA-infected motoneurons and uninfected and mock-infected controls stimulated for two (**c**) or five (**d**) minutes with BDNF. No differences in Akt phosphorylation at Thr^308^ (*left blots*) and Ser^473^ (*right blots*) were observed after *Syap1* knockdown compared to controls. The detection of Syap1 (*right blots*) demonstrates the strong reduction in Syap1 protein levels by the knockdown. Quantification of the signals of these and similar *blots* is shown in Fig. S8

**Fig. 14:**
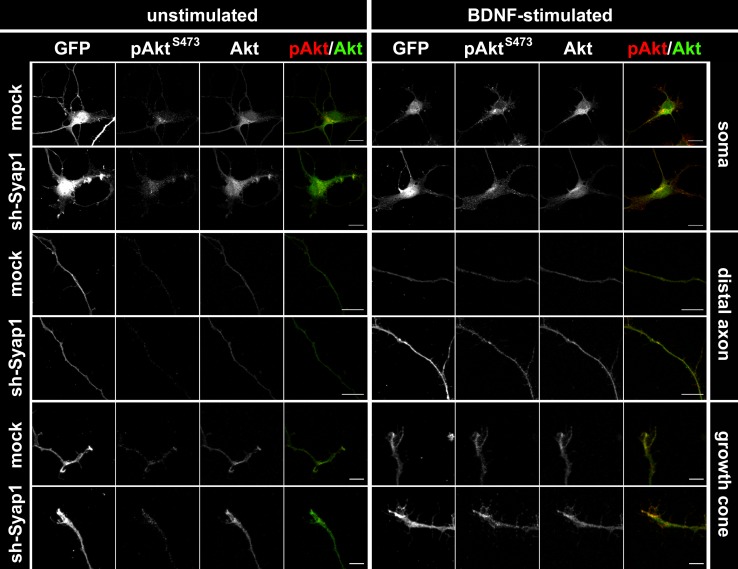
pAkt^Ser473^/Akt ratios are not altered in different compartments of stimulated motoneurons. After 5 DIV in the presence of CNTF (5 ng/ml), the cells were serum as well as neurotrophic factor deprived for 6–7 h and stimulated for 5 min with BDNF (20 ng/ml), followed by fixation and staining against pAkt^Ser473^, Akt, and GFP. Compartment-specific quantification of the pAkt^Ser473^/Akt ratios is shown in Fig. S9. pAkt^Ser473^/Akt ratios strongly increase upon BDNF stimulation, but comparable pAkt^Ser473^/Akt values were achieved after *Syap1* knockdown or mock treatment in all the tested compartments of the motoneurons. *Scale bars* Soma and axon: 10 µm; growth cone: 5 µm

In adipocyte differentiation, Syap1 regulates growth factor-induced Akt1 phosphorylation at Ser^473^, and *Syap1* knockdown by shRNA strongly reduces this phosphorylation (Yao et al. 2013). To investigate whether knockdown or knockout of *Syap1* in motoneurons also causes reduced Akt phosphorylation, we stimulated primary motoneurons after 5 DIV in the presence of CNTF (5 ng/ml) and serum, followed by neurotrophic factor deprivation for variable times (see Methods), with BDNF (20 ng/ml) and tested the levels of Akt phosphorylation at Thr^308^ and Ser^473^ using phospho-specific antisera. In order to determine Akt phosphorylation kinetics at Thr^308^ and Ser^473^, we first stimulated motoneurons for increasing durations ranging from 2 s to 30 min. Maximal Akt phosphorylation was achieved after 2–5 min of stimulation for both sites (Thr^308^ and Ser^473^) (Fig. 13a, *n* = 3). Since localization data were identical for knock-out and knock-down motoneurons (Fig. 10), and Western blots confirmed almost complete Syap1 deficiency in knock-down neurons (Fig. 13c, d right panels), the effect of Syap1 deficiency on Akt phosphorylation was determined with knock-out neurons after 5 min of BDNF stimulation (Fig. 13b), whereas experiments after 2- and 5-min stimulation (Figs. 13c, d, S8) and tests for subcompartimental differences in stimulation effects (Figs. 14, S9) were carried out on knock-down neurons which could be generated in comparatively large quantities from motoneurons pooled from wild-type embryos. The Western blots of Fig. 13 revealed no reduction in Akt phosphorylation at Thr^308^ or Ser^473^ upon *Syap1* knockout or knockdown compared to the controls. For the quantitative evaluation, densitometric signal values were normalized to the stimulated uninfected controls. The results depicted in Fig. S8 show no significant difference in Akt phosphorylation after *Syap1* knockout (Fig. S8a, 5-min stimulation, values normalized to stimulated wild type; Thr^308^(left): unstimulated, wild type 0.13 ± 0.06; unstimulated, knockout: 0.15 ± 0.06; stimulated, knockout: 1.58 ± 0.06 (*n* = 3), Ser^473^ (right) unstimulated, wild type 0.23 ± 0.06; unstimulated, knockout: 0.24 ± 0.08; stimulated, knockout: 1.48 ± 0.29 (*n* = 3)) or knockdown (Fig. S8b, values normalized to uninfected stimulated pAkt/Akt ratio, 2-min stimulation; Thr^308^(left): unstimulated, uninfected: 0.12 ± 0.06; stimulated, *sh*-*Syap1*-infected: 1.58 ± 0.16; stimulated, mock-infected: 1.27 ± 0.26 (*n* = 3), Ser^473^ (right) unstimulated, uninfected: 0.39 ± 0.05; stimulated, *sh*-*Syap1*-infected: 1.27 ± 0.13, stimulated, mock-infected: 1.08 ± 0.04 (*n* = 3)); Fig. S8c, 5-min stimulation; Thr^308^ (left): unstimulated, uninfected: 0.06 ± 0.03; stimulated, *sh*-*Syap1*-infected: 1.31 ± 0.15; stimulated, mock-infected: 0.92 ± 0.05 (*n* = 3); Ser^473^ (right) unstimulated, uninfected: 0.23 ± 0.07; stimulated, *sh*-*Syap1*-infected: 1.72 ± 0.17; stimulated, mock-infected: 1.54 ± 0.24 (*n* = 3)).

In contrast to fibroblasts or adipocytes, neurons are forming different cellular compartments such as soma, axon, and growth cones. Thus, Syap1 might mediate *local* changes in Akt phosphorylation in these compartments. Such local changes in Akt phosphorylation might escape detection when whole cells are lysed for Western blot analysis. In order to investigate whether neuronal Syap1 inhibits Akt phosphorylation specifically in cell bodies, axons, or growth cones, we used Akt- and pAkt^Ser473^-specific antibodies on cultured motoneurons to immunocytochemically determine phosphorylation of Akt at Ser^473^ separately in these compartments under conditions of 5 days in culture, 6- to 7-h starvation, and 5-min BDNF stimulation (20 ng/ml) (Fig. 14). Quantification of immunocytochemical fluorescence signals in *Syap1* shRNA- or mock-treated primary motoneurons shows that the ratio of pAkt^Ser473^ to total Akt does not decrease after *Syap1* knockdown, irrespective of whether signals from soma, distal axons, or axonal growth cones are analyzed (Fig. S9) (pAkt^Ser473^/Akt ratios normalized to unstimulated mock-infected condition: soma: unstimulated *sh*-*Syap1*-infected: 0.92 ± 0.09; stimulated, mock-infected: 4.85 ± 1.07; stimulated, *sh*-*Syap1*-infected: 4.46 ± 0.86; distal axon: unstimulated *sh*-*Syap1*-infected: 0.90 ± 0.07; stimulated, mock-infected: 3.11 ± 0.51; stimulated *sh*-*Syap1*-infected: 3.28 ± 0.55; growth cone: unstimulated, *sh*-*Syap1*-infected: 0.86 ± 0.04; stimulated, mock-infected: 3.01 ± 0.60; stimulated, *sh*-*Syap1*-infected: 3.25 ± 0.70, *n* = 5 experiments, total number of cells evaluated per condition: 78–93). Likewise, no significant effect of *Syap1* knockdown on pAkt^Ser473^/Akt ratios normalized to mock controls is seen when motoneurons were cultured under standard conditions without starvation and stimulation (Fig. S9b). These findings support the conclusion from the Western blots of cell lysates (Figs. 13c, d, S8) that *Syap1* knockdown in motoneurons does not decrease Akt phosphorylation at Ser^473^ and demonstrates that there are apparently no compartment-specific effects regarding this phosphorylation in somata, axons, or growth cones.

## Discussion

Syap1 and Sap47 represent a protein family of unknown molecular function in the brain, but since its gene has been conserved from *C. elegans* and *Drosophila* to mouse and human, it is expected to convey a selection advantage. With the aim to determine its cellular and systemic distribution and function in the nervous system of mammals, we have generated a knock-out mouse and show here that the *tm1a* mutation of the *Syap1* gene (Fig. 1) eliminates detectible amounts of Syap1 protein. In the knock-out brain, 6 % (14 %) of wild-type levels of *Syap1* transcripts containing exon-3 and exon-4 (exon-8 and exon-9) are observed, but no wild-type protein can be detected (Figs. 2, S2b). This discrepancy between transcript and protein level reductions in the mutant could perhaps be explained by competition for translation initiation of the short transcripts containing exon-1 to exon-3 and *En*-*2*-*lacZ* sequences with the residual intact *Syap1* transcripts. The different amounts of transcripts containing exon-3 and exon-4 compared to exon-8 and exon-9 might be due to partial RNA degradation or independent transcription initiation between exon-4 and exon-8.

Syap1 is found in most tissues of the adult mouse including nervous system (Figs. 2b, S2a). In the mouse brain, Syap1 immunoreactivity is widely distributed. Relatively high levels of Syap1 are found in neuropil regions with high numbers of glutamatergic terminals, such as the hippocampal mossy fiber pathway (Kohara et al. 2014) (Figs. 6, S5a, b) and the molecular layer of the cerebellum (Figs. 7, S5c). Non-neural cells in the brain appear to contain only low amounts of Syap1 (Figs. 5, S4a, b). Also, the nerve layer of the olfactory bulb and the olfactory glomeruli, containing the axons and the glutamatergic terminals of the olfactory sensory neurons, respectively (Ennis et al. 2006), are rich in Syap1 (Fig. 4). In the cortex, the distribution of Syap1 seems to be more homogeneous, although again patterns of higher levels can be recognized which do not match with patterns of high density of somata as revealed by nuclear DAPI staining and thus may represent synaptic accumulations (Figs. 3a, S3). The apparently neuron-subtype-specific selective perinuclear Syap1 localization (in most but not all neuronal somata in a subdomain of the thalamus; in CA2, and with lower abundance in CA1 or CA3 hippocampal pyramidal cells) indicates that *Syap1* is not simply a housekeeping gene but must have a function specific for a given cell type. Syap1 is also found in perinuclear structures of cholinergic neurons in the spinal cord, but it does not accumulate in cholinergic synaptic boutons on these cells (Fig. 8). Low concentrations of Syap1 are observed at the cholinergic neuromuscular junctions, apparently both presynaptically and in muscle tissue (Fig. 9).

As tentative résumé of this initial characterization of the distribution of Syap1 in the mouse central nervous system, we propose that Syap1 is present in most neuropil regions with high concentrations in regions containing glutamatergic synaptic terminals where vGlut1 co-labeling suggests that it may well be presynaptically localized, but that it may also be present in postsynaptic structures (Figs. 7, S5c). Its conspicuous perinuclear accumulation near the Golgi complex appears to be prominent in specific neuronal cell types, among which are glutamatergic (e.g., CA2 pyramidal cells), GABAergic (e.g., Purkinje cells), and cholinergic neurons (spinal cord motoneurons). However, the elimination of Syap1 from these cells does not obviously alter the fine structure of the Golgi complex as seen in confocal microscopy (Fig. S6). The association of Syap1 with the Golgi complex apparently is not limited to nervous system cells, as suggested by Syap1 and GM130 double labeling of human embryonic kidney cells (Fig. S7). The ultrastructural and molecular basis of this association and the role of Syap1 in the complex functions of the Golgi organelle now need to be determined.

In addition to describing the distribution of Syap1 in the mouse brain, we tested whether this protein could play a major role in early motoneuron differentiation and cell-autonomous axon growth and growth cone development in cultured embryonic motoneurons. This was of particular interest in view of the importance of Syap1 for the differentiation of adipocytes from murine embryonic stem cells (Yao et al. 2013). In isolated mouse primary motoneurons, Syap1 is concentrated in a perinuclear region but is also present in axonal compartments (Fig. 10). Syap1 is detected in the axonal shaft as well as in the filopodia of the growth cone (Fig. 10e, k). This again indicates that Syap1 could have a presynaptic function similar to what was shown for Sap47 in *Drosophila* (Reichmuth et al. 1995; Saumweber et al. 2011).

In the motoneuron soma, Syap1 was found in close proximity to the *cis/medial*-Golgi marker GM130 and a component of the AP1 complex. The AP1 complex is one of four distinct AP complexes (AP1–4) which are present in mammalian cells, and each is reported to be implicated in a different trafficking pathway (Kirchhausen 1999; Robinson and Bonifacino 2001). Besides its involvement in the anterograde transport from the *trans*-Golgi network (TGN) to endosomes, the AP1 complex is also believed to have a role in the recycling from endosomes to the TGN (Ahle et al. 1988; Robinson 1990; Meyer et al. 2000, 2001). The spatial relation of Syap1 with the *cis*-Golgi and the AP1 complex was investigated by super-resolution microscopy (Fig. 11e–h). We conclude that Syap1 apparently does not co-localize with either GM130 or AP1 as the overlap with Syap1 signals decreases in super-resolution microscopy compared to normal confocal imaging.

The assumption of a synaptic role of Syap1, as its name suggests, was mainly based on the characterization of the *Drosophila* Syap1 homologue Sap47 which is predominantly found in synaptic neuropil and associated with synaptic vesicles in glutamatergic synaptic boutons of larval motoneurons. The fact that in *Drosophila* larvae the Sap47 gene is required for normal synaptic plasticity and associative learning/memory (Saumweber et al. 2011) supports this assumption. However, developmental expression data for *Drosophila* (flybase, (St Pierre et al. 2014)) indicate that Sap47 transcription is already observed in the early embryo and thus is not linked to synaptogenesis, suggesting a more general cellular function. The prominent localization of Syap1 in perinuclear Golgi-associated structures and in regions rich in glutamatergic synaptic terminals described here is consistent with both a synaptic and a more general cellular function also in mammals.

For adipocytes, it has been shown that Syap1 promotes mTORC2-mediated phosphorylation of Akt1 at Ser^473^ upon growth factor stimulation, and that this Syap1–Akt1 interaction is critical for in vitro differentiation of these cells (Yao et al. 2013). In neurons, activation of Akt can be induced by neurotrophins such as BDNF (Huang and Reichardt 2003). Akt is phosphorylated at various amino acids. The protein is constitutively phosphorylated at a highly conserved site (Thr^450^) in the turn motive directly after translation, facilitating its folding and stability (Facchinetti et al. 2008). Akt is then fully activated by a dual phosphorylation at Thr^308^ and Ser^473^ (Alessi et al. 1996; Anderson et al. 1998; Sarbassov et al. 2005; Jacinto et al. 2006). In the mammalian nervous system, all three Akt genes are expressed. Phenotypes of single and double knock-out mice suggest that the functions of the three isoforms are only partially redundant (Diez et al. 2012; Cohen et al. 2011; Hers et al. 2011). The three proteins are so similar that the phospho-specific antibodies used in this study detect all three isoforms. Akt3 has been shown to be of particular importance in the nervous system (Tschopp et al. 2005; Easton et al. 2005). Thus, we cannot exclude that a possible Akt1 hypophosphorylation in motoneurons might be masked, e.g., by Akt3 hyperphosphorylation. The lack of changes in growth factor-induced Akt phosphorylation upon *Syap1* knockout or knockdown in NSC cells (not shown) or in BDNF-stimulated or BDNF-unstimulated primary motoneurons (Fig. 13b–d) does not support the hypothesis of a functionally important Syap1–Akt interaction in these cells. Our experiments show that the knockdown of *Syap1* (Fig. 13c, d, quantified in Fig. S1e) or its knockout (Fig. 2) apparently does not induce alterations in total Akt phosphorylation, neither in Western blots of homogenized motoneurons nor in immunocytochemical analysis of soma, axon, or growth cone, irrespective of the stimulation regime (Figs. 13, 14, S8, S9). We therefore propose that Syap1 may be involved in different molecular functions depending on cell type and perhaps developmental state, and we speculate that Syap1 function in motoneurons is either independent of the Akt signaling pathway or isoform-specific mechanisms in the nervous system operate to keep total Akt phosphorylation constant.

Altogether, considering the association of Syap1 with the Golgi complex shown here and the proximity of Sap47 to glutamatergic synaptic vesicles in *Drosophila*, our data are compatible with a hypothesis that in the nervous system Syap1/Sap47 could be involved in vesicular trafficking in a large subpopulation of cell somata and in certain types of synaptic terminals. The function of this protein family, however, appears rather subtle as indicated by the viability of the knock-out flies and mice and the fact that in cultured mouse motoneurons no effects of *Syap1* knockout on survival or axon outgrowth could be detected (Fig. 12). The characterization of the behavioral phenotype of the *Syap1* knock-out mouse is therefore eagerly awaited.

## Electronic supplementary material

Below is the link to the electronic supplementary material.

Supplementary material 1 (DOCX 7697 kb)

## References

[CR1] Ahle S, Mann A, Eichelsbacher U, Ungewickell E (1988). Structural relationships between clathrin assembly proteins from the Golgi and the plasma membrane. EMBO J.

[CR2] Al-Dhaheri MH, Shah YM, Basrur V, Pind S, Rowan BG (2006). Identification of novel proteins induced by estradiol, 4-hydroxytamoxifen and acolbifene in T47D breast cancer cells. Steroids.

[CR3] Alessi DR, Andjelkovic M, Caudwell B, Cron P, Morrice N, Cohen P, Hemmings BA (1996). Mechanism of activation of protein kinase B by insulin and IGF-1. EMBO J.

[CR4] Anderson KE, Coadwell J, Stephens LR, Hawkins PT (1998). Translocation of PDK-1 to the plasma membrane is important in allowing PDK-1 to activate protein kinase B. Curr Biol.

[CR5] Arakawa Y, Sendtner M, Thoenen H (1990). Survival effect of ciliary neurotrophic factor (CNTF) on chick embryonic motoneurons in culture: comparison with other neurotrophic factors and cytokines. J Neurosci.

[CR6] Boehm M, Bonifacino JS (2001). Adaptins: the final recount. Mol Biol Cell.

[CR7] Bolte S, Cordelieres FP (2006). A guided tour into subcellular colocalization analysis in light microscopy. J Microsc.

[CR8] Briese M, Saal L, Appenzeller S, Moradi M, Baluapuri A, Sendtner M (2016). Whole transcriptome profiling reveals the RNA content of motor axons. Nucleic Acids Res.

[CR9] Calof AL, Reichardt LF (1984). Motoneurons purified by cell sorting respond to two distinct activities in myotube-conditioned medium. Dev Biol.

[CR10] Chang YC, Yu YL, Wang N, Xu YH (2001). Cloning and characterization of Syap1, a down regulated gene in human hepatocellular carcinoma. Shi yan sheng wu xue bao.

[CR11] Cohen Y, Goldenberg-Cohen N, Shalmon B, Shani T, Oren S, Amariglio N, Dratviman-Storobinsky O, Shnaiderman-Shapiro A, Yahalom R, Kaplan I, Hirshberg A (2011). Mutational analysis of PTEN/PIK3CA/AKT pathway in oral squamous cell carcinoma. Oral Oncol.

[CR12] Cooper JA (1987). Effects of cytochalasin and phalloidin on actin. J Cell Biol.

[CR13] Costes SV, Daelemans D, Cho EH, Dobbin Z, Pavlakis G, Lockett S (2004). Automatic and quantitative measurement of protein-protein colocalization in live cells. Biophys J.

[CR14] Cox BJ, Vollmer M, Tamplin O, Lu M, Biechele S, Gertsenstein M, van Campenhout C, Floss T, Kuhn R, Wurst W, Lickert H, Rossant J (2010). Phenotypic annotation of the mouse X chromosome. Genome Res.

[CR15] Diez H, Garrido JJ, Wandosell F (2012). Specific roles of Akt iso forms in apoptosis and axon growth regulation in neurons. PLoS One.

[CR16] Doerks T, Huber S, Buchner E, Bork P (2002). BSD: a novel domain in transcription factors and synapse-associated proteins. Trends Biochem Sci.

[CR17] Easton RM, Cho H, Roovers K, Shineman DW, Mizrahi M, Forman MS, Lee VM, Szabolcs M, de Jong R, Oltersdorf T, Ludwig T, Efstratiadis A, Birnbaum MJ (2005). Role for Akt3/protein kinase B*γ* in attainment of normal brain size. Mol Cell Biol.

[CR18] Ennis M, Zhu M, Heinbockel T, Hayar H (2006). Olfactory nerve–evoked, metabotropic glutamate receptor–mediated synaptic responses in rat olfactory bulb mitral cells. J Neurophysiol.

[CR19] Facchinetti V, Ouyang W, Wei H, Soto N, Lazorchak A, Gould C, Lowry C, Newton AC, Mao Y, Miao RQ, Sessa WC, Qin J, Zhang P, Su B, Jacinto E (2008). The mammalian target of rapamycin complex 2 controls folding and stability of Akt and protein kinase C. EMBOJ.

[CR20] Forscher P, Smith SJ (1988). Actions of cytochalasins on the organization of actin filaments and microtubules in a neuronal growth cone. J Cell Biol.

[CR21] Funk N, Becker S, Huber S, Brunner M, Buchner E (2004). Targeted mutagenesis of the Sap47 gene of *Drosophila*: flies lacking the synapse associated protein of 47 kDa are viable and fertile. BMC Neurosci.

[CR22] Hers I, Vincent EE, Tavare JM (2011). Akt signalling in health and disease. Cell Signal.

[CR23] Hofbauer A, Ebel T, Waltenspiel B, Oswald P, Chen YC, Halder P, Biskup S, Lewandrowski U, Winkler C, Sickmann A, Buchner S, Buchner E (2009). The Würzburg hybridoma library against *Drosophila* brain. J Neurogenet.

[CR24] Huang EJ, Reichardt LF (2003). Trk receptors: roles in neuronal signal transduction. Ann Rev Biochem.

[CR25] Jablonka S, Beck M, Lechner BD, Mayer C, Sendtner M (2007). Defective Ca2+ channel clustering in axon terminals disturbs excitability in motoneurons in spinal muscular atrophy. J Cell Biol.

[CR26] Jacinto E, Facchinetti V, Liu D, Soto N, Wei S, Jung SY, Huang Q, Qin J, Su B (2006). SIN1/MIP1 maintains rictor-mTOR complex integrity and regulates Akt phosphorylation and substrate specificity. Cell.

[CR27] Kirchhausen T (1999). Adaptors for clathrin-mediated traffic. Ann Rev Cell Dev Biol.

[CR28] Kohara K, Pignatelli M, Rivest AJ, Jung HY, Kitamura T, Suh J, Frank D, Kajikawa K, Mise N, Obata Y, Wickersham IR, Tonegawa S (2014). Cell type-specific genetic and optogenetic tools reveal hippocampal CA2 circuits. Nat Neurosci.

[CR29] Meyer C, Zizioli D, Lausmann S, Eskelinen EL, Hamann J, Saftig P, von Figura K, Schu P (2000). mu1A-adaptin-deficient mice: lethality, loss of AP-1 binding and rerouting of mannose 6-phosphate receptors. EMBO J.

[CR30] Meyer C, Eskelinen EL, Guruprasad MR, von Figura K, Schu P (2001). Mu 1A deficiency induces a profound increase in MPR300/IGF-II receptor internalization rate. J Cell Sci.

[CR31] Nakamura N, Rabouille C, Watson R, Nilsson T, Hui N, Slusarewicz P, Kreis TE, Warren G (1995). Characterization of a *cis*-Golgi matrix protein, GM130. J Cell Biol.

[CR32] Paglini G, Pigino G, Kunda P, Morfini G, Maccioni R, Quiroga S, Ferreira A, Caceres A (1998). Evidence for the participation of the neuron-specific CDK5 activator P35 during laminin-enhanced axonal growth. J Neurosci.

[CR33] Reichmuth C, Becker S, Benz M, Debel K, Reisch D, Heimbeck G, Hofbauer A, Klagges B, Pflugfelder GO, Buchner E (1995). The sap47 gene of *Drosophila* melanogaster codes for a novel conserved neuronal protein associated with synaptic terminals. Brain Res.

[CR34] Robinson MS (1990). Cloning and expression of *γ*-adaptin, a component of clathrin-coated vesicles associated with the Golgi apparatus. J Cell Biol.

[CR35] Robinson MS, Bonifacino JS (2001). Adaptor-related proteins. Curr Opin Cell Biol.

[CR36] Rogers ML, Atmosukarto I, Berhanu DA, Matusica D, Macardle P, Rush RA (2006). Functional monoclonal antibodies to p75 neurotrophin receptor raised in knockout mice. J Neurosci Methods.

[CR37] Rossoll W, Jablonka S, Andreassi C, Kroning AK, Karle K, Monani UR, Sendtner M (2003). Smn, the spinal muscular atrophy-determining gene product, modulates axon growth and localization of *β*-actin mRNA in growth cones of motoneurons. J Cell Biol.

[CR38] Sanes JR, Lichtman JW (1999). Development of the vertebrate neuromuscular junction. Ann Rev Neurosci.

[CR39] Sarbassov DD, Guertin DA, Ali SM, Sabatini DM (2005). Phosphorylation and regulation of Akt/PKB by the rictor-mTOR complex. Science.

[CR40] Saumweber T, Weyhersmuller A, Hallermann S, Diegelmann S, Michels B, Bucher D, Funk N, Reisch D, Krohne G, Wegener S, Buchner E, Gerber B (2011). Behavioral and synaptic plasticity are impaired upon lack of the synaptic protein SAP47. J Neurosci.

[CR41] Schermelleh L, Heintzmann R, Leonhardt H (2010). A guide to super-resolution fluorescence microscopy. J Cell Biol.

[CR42] Selvaraj BT, Frank N, Bender FL, Asan E, Sendtner M (2012). Local axonal function of STAT3 rescues axon degeneration in the pmn model of motoneuron disease. J Cell Biol.

[CR43] Sendtner M, Pei G, Beck M, Schweizer U, Wiese S (2000). Developmental motoneuron cell death and neurotrophic factors. Cell Tissue Res.

[CR44] Skarnes WC, Rosen B, West AP, Koutsourakis M, Bushell W, Iyer V, Mujica AO, Thomas M, Harrow J, Cox T, Jackson D, Severin J, Biggs P, Fu J, Nefedov M, de Jong PJ, Stewart AF, Bradley A (2011). A conditional knockout resource for the genome-wide study of mouse gene function. Nature.

[CR45] St Pierre SE, Ponting L, Stefancsik R, McQuilton P, FlyBase C (2014). FlyBase 102—advanced approaches to interrogating FlyBase. Nucleic Acids Res.

[CR46] Subramanian N, Wetzel A, Dombert B, Yadav P, Havlicek S, Jablonka S, Nassar MA, Blum R, Sendtner M (2012). Role of Na(v)1.9 in activity-dependent axon growth in motoneurons. Hum Mol Genet.

[CR47] Tschopp O, Yang ZZ, Brodbeck D, Dummler BA, Hemmings-Mieszczak M, Watanabe T, Michaelis T, Frahm J, Hemmings BA (2005). Essential role of protein kinase B*γ* (PKB*γ*/Akt3) in postnatal brain development but not in glucose homeostasis. Development.

[CR48] Wiese S, Metzger F, Holtmann B, Sendtner M (1999). The role of p75NTR in modulating neurotrophin survival effects in developing motoneurons. Eur J Neurosci.

[CR49] Wiese S, Herrmann T, Drepper C, Jablonka S, Funk N, Klausmeyer A, Rogers ML, Rush R, Sendtner M (2010). Isolation and enrichment of embryonic mouse motoneurons from the lumbar spinal cord of individual mouse embryos. Nat Protoc.

[CR50] Yao Y, Suraokar M, Darnay BG, Hollier BG, Shaiken TE, Asano T, Chen CH, Chang BH, Lu Y, Mills GB, Sarbassov D, Mani SA, Abbruzzese JL, Reddy SA (2013). BSTA promotes mTORC2-mediated phosphorylation of Akt1 to suppress expression of FoxC2 and stimulate adipocyte differentiation. Sci Signal.

