# Supplementary material for: Initial characterization of a Syap1 knock-out mouse and distribution of Syap1 in mouse brain and cultured motoneurons
Source: Histochem Cell Biol. 2016 Jun 25;146(4):489–512. doi: 10.1007/s00418-016-1457-0 (PMC5037158; doi:10.1007/s00418-016-1457-0)
Supplement: Supplementary file 1 — Supplementary material 1 (DOCX 7697 kb) [file 418_2016_1457_MOESM1_ESM.docx]

**Online Resource 1 as Supplement to**

Initial characterization of a *Syap1* knock-out mouse and distribution of Syap1 in mouse brain and cultured motoneurons

Histochemistry and Cell Biology

Dominique Schmitt, Natalia Funk, Robert Blum, Esther Asan, Lill Andersen, Thomas Rülicke, Michael Sendtner, Erich Buchner

Corresponding author:

Erich Buchner, Institute for Clinical Neurobiology, Versbacher Str. 5, 97078 Würzburg, Germany (email: [Buchner_E@ukw.de](mailto:Buchner_E@ukw.de))

**Verification of an anti-Syap1 antiserum and Syap1 expression in nerves and muscle**

We tested the specificity of the antiserum against the full-length human Syap1 protein (16272-1AP Proteintech) that was used in the present study. Protein lysates from human embryonic kidney cells (HEK293T) and from mouse motor neuron-like hybrid cells (NSC34) were analyzed by Western blots. We observed one prominent band for each cell line and a small species-specific difference in electrophoretic mobility, consistent with the fact that mouse Syap1 (RefSeq: NM_025932) is 13 amino acids longer than its human autologue (RefSeq: NM_032796) (Fig. S1a). The bands recognized migrate at 55 - 56 kDa, significantly higher than predicted by the calculated molecular weight of approximately 40 kDa (mouse: 41.36 kDa; human 39.94 kDa). Thus, similar to Sap47 in *Drosophila*, mammalian Syap1 shows anomalous electrophoretic mobility which in *Drosophila* is not due to posttranslational modification, as has been shown by bacterial expression of cDNA (Reichmuth *et al.*, 1995).

When a FLAG-tagged mouse Syap1 was over-expressed in HEK293T cells, two bands were detected by the antibody in Western blots. The endogenous protein was observed at 55 kDa and the band for the tagged protein at approximately 60 kDa (Fig. S1b). This clearly indicates that the antiserum binds to both murine and human Syap1 with high selectivity. We used this antibody to determine the efficacy of a lentiviral shRNA *Syap1* knock-down vector in NSC34 cells and cultured primary motoneurons. For both cell lysates the detected band at 56 kDa was strongly reduced when the cells had been infected with the *Syap1* shRNA virus (knock-down) whereas the signals of the mismatch shRNA virus (“mock”) treated and uninfected controls remained unaffected (Fig. S1c and d). Both lentiviruses (mock and *Syap1* knock-down) expressed GFP as a reporter for successful infection. The lentiviral knock-down efficacy for Syap1 protein levels was determined with primary motoneurons cultures. In comparison to uninfected or mock-infected motoneurons, *Syap1* knock-down virus reduced anti-Syap1 immunoreactivity by 91 ± 1.8 %, as analyzed by densitometric measurements of Western blots (Fig. S1e). These results validate the specificity of this antibody for detection of human and mouse Syap1 protein in Western Blots. We also show that Syap1 is expressed in nerves and muscles (Fig. S2a).


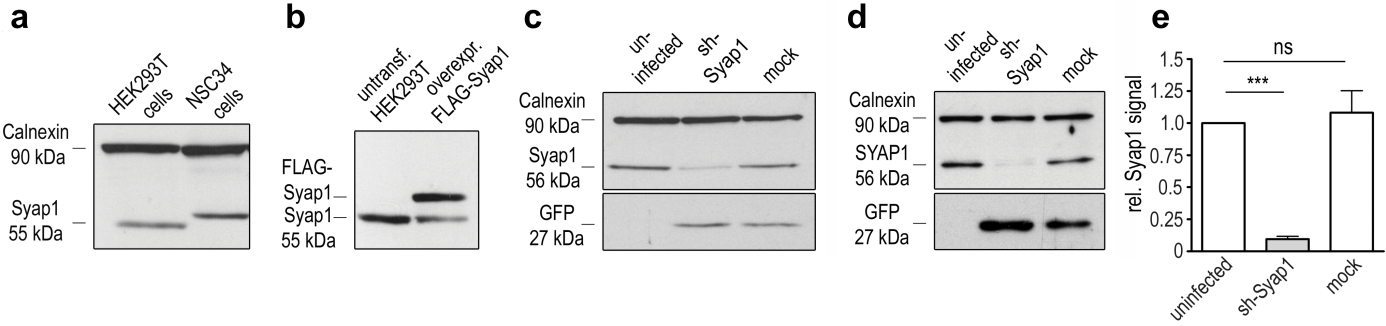


Figure S1

**Fig. S1** Validation of anti-Syap1 antibody specificity and efficiency of *Syap1* knock-down in Western blot. (**a**) In HEK293T cells the antibody against human Syap1 detects a protein band at 55 kDa and in mouse NSC34 cells at 56 kDa, indicating that this antibody also cross-reacts with the murine Syap1 protein which is 13 amino acids larger than human Syap1. (**b**) Overexpression of FLAG-tagged mouse Syap1 in HEK293T cells leads to antibody signals of the endogenous as well as the over-expressed protein. Lentiviral *Syap1* knock-down strongly reduces the signal in NSC cells and primary motoneurons (**c**, **d**). Calnexin served as loading control, GFP signals demonstrate successful lentiviral infection. (**e**) Quantification of the *Syap1* knock-down efficacy by densitometric analysis from Western blots. Syap1/Calnexin ratios were determined and normalized to the uninfected control. A reduction of 91 ± 1.8 % of Syap1 protein is achieved in cultured primary motoneurons after 5 DIV (n = 8). ***: *P* < 0.001; ns = not significant: *P* > 0.05





Figure S2

**Fig. S2** (**a**) Syap1 is also expressed in nerves and muscle. Blot similar to Fig. 2b (left half) including additional tissues such as nerves and muscle. (**b**) Syap1 protein levels in the *Syap1^tm1a^* mutant are reduced to less than 1% of wildtype levels. No trace of Syap1 is detected in mutant hippocampus lysate (lane 1), whereas the corresponding wildtype lysate generates a discernible signal at 1:100 dilution (arrow). Lanes: 1, mutant; 2, wildtype undiluted; 3, wildtype diluted 1:10; 4, wildtype diluted 1:30; 5, wildtype diluted 1:100

**The Syap1 antiserum cross-reacts with unknown structures in the mouse brain**


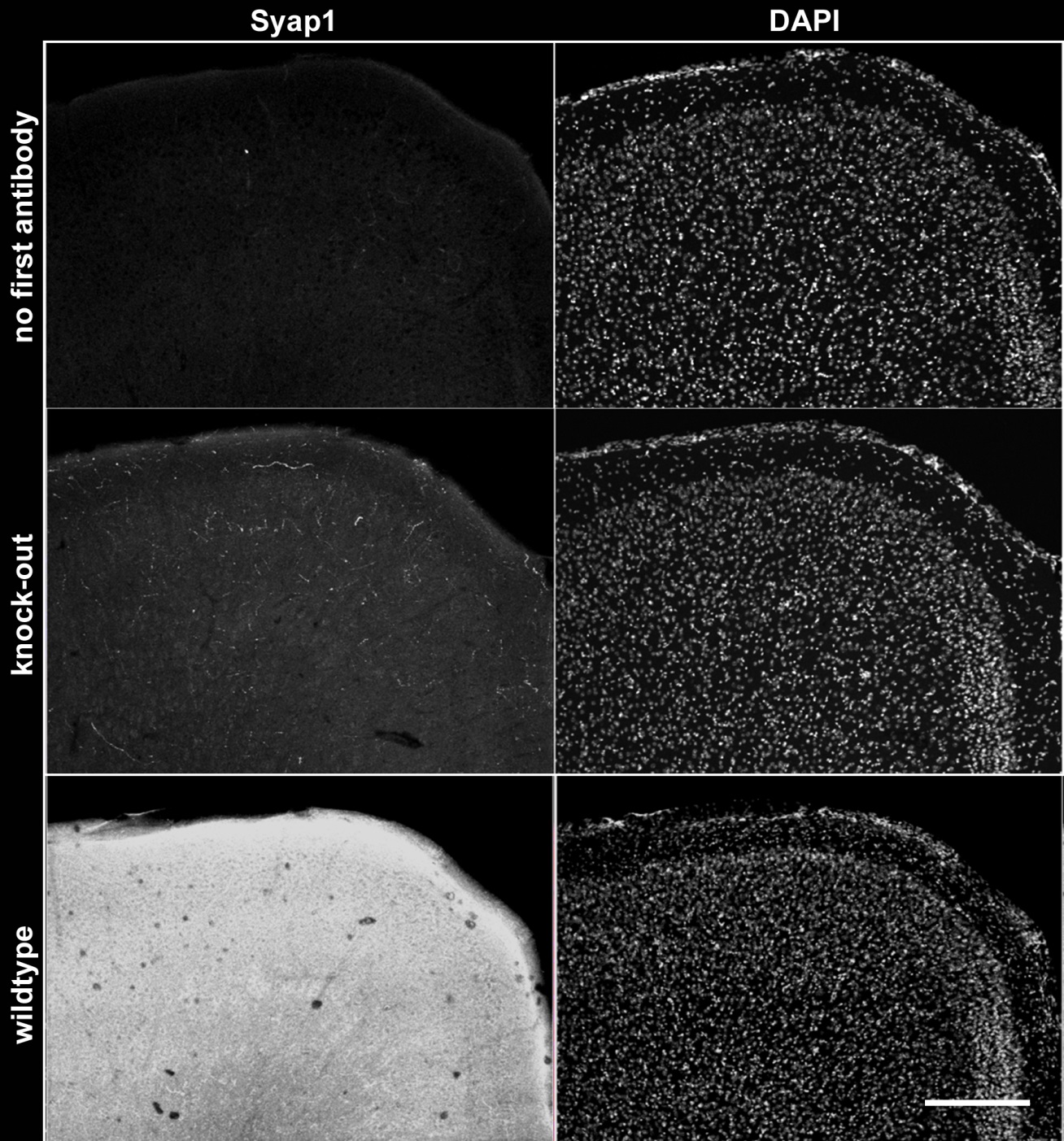


Figure S3

**Fig. S3** Control staining of wildtype sections without first antibody (top left) reveals structures that are stained by the Syap1 antiserum due to unspecific crossreaction as seen in *Syap1* knock-out sections (middle left), exemplified here for the dorso-medial cerebral cortex. In wildtype sections these structures are also observed but are often outshone by the specific Syap1 staining (bottom left). Right: Nuclear counterstaining by DAPI. Scale bar: 250 µm

**Syap1 is strongly expressed in many neurons of the thalamus but is almost absent in astrocytes**


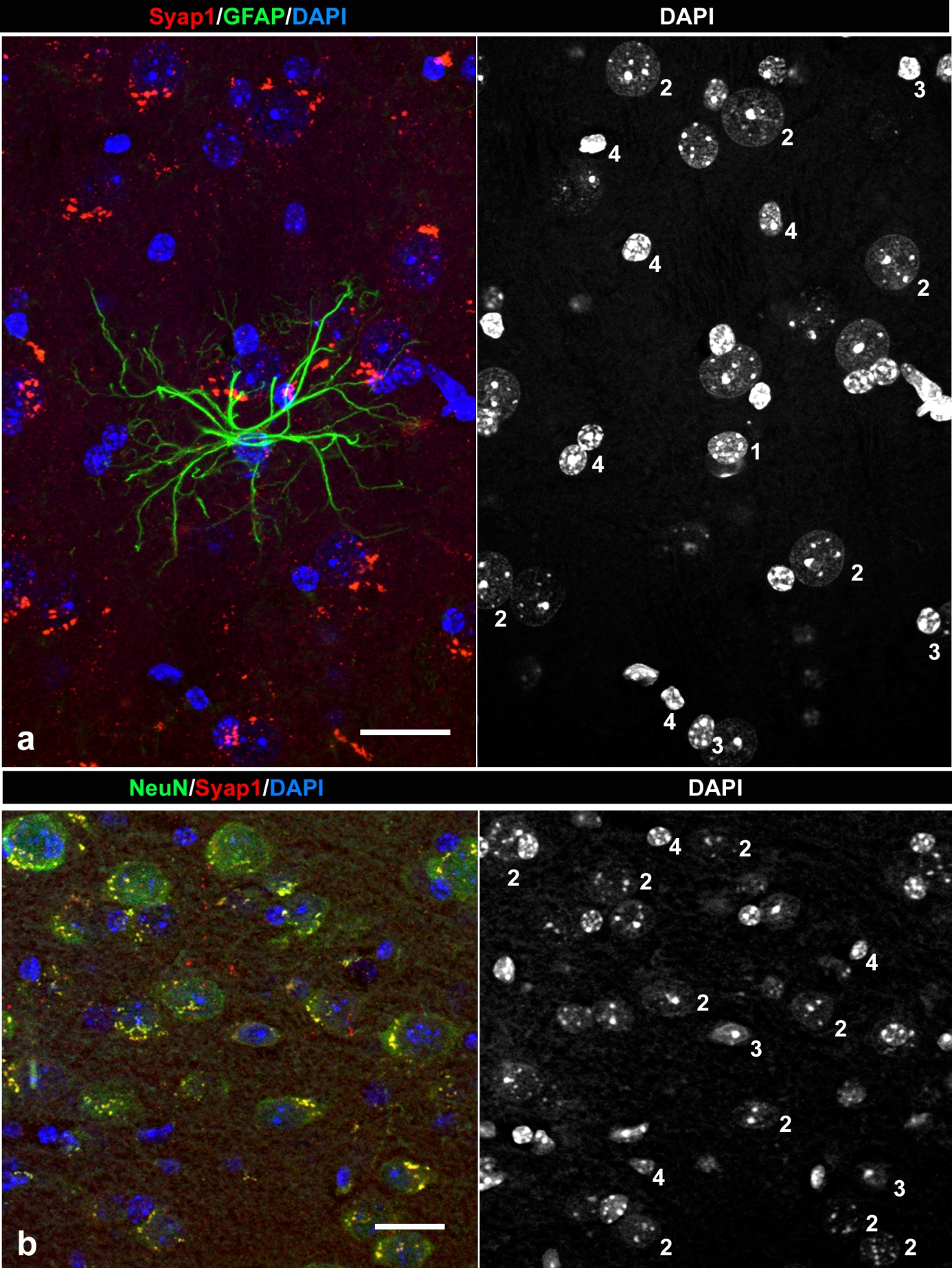


Figure S4

**Fig. S4 a**) Example of an astrocyte labeled with anti-GFAP (green) in the thalamus, with its typical nucleus labeled “1” in the nuclear staining (right). The astrocyte almost completely lacks Syap1 IR (red) while most nuclei with typical characteristics of neuronal muclei (large, euchromatin-rich, marked “2”) are surrounded by strong cytoplasmic Syap1 IR (associated with Golgi marker, see Fig. 5 main text). Occasional Syap1 IR observed near smaller nuclei (marked “3”) may actually be associated with large nuclei localized in their immediate vicinity. Most small nuclei are not detectably associated with Syap1 IR (marked “4”). **b**) Double staining of a thalamic region with the neuronal marker anti-NeuN (green) and anti-Syap1 (red) demonstrates that most neurons in this region contain Syap1 IR (marking of nuclei as in **a**). Scale bars 20 µm

**Syap1 IR is present in glutamatergic synaptic terminals but also in adjacent neuropil**


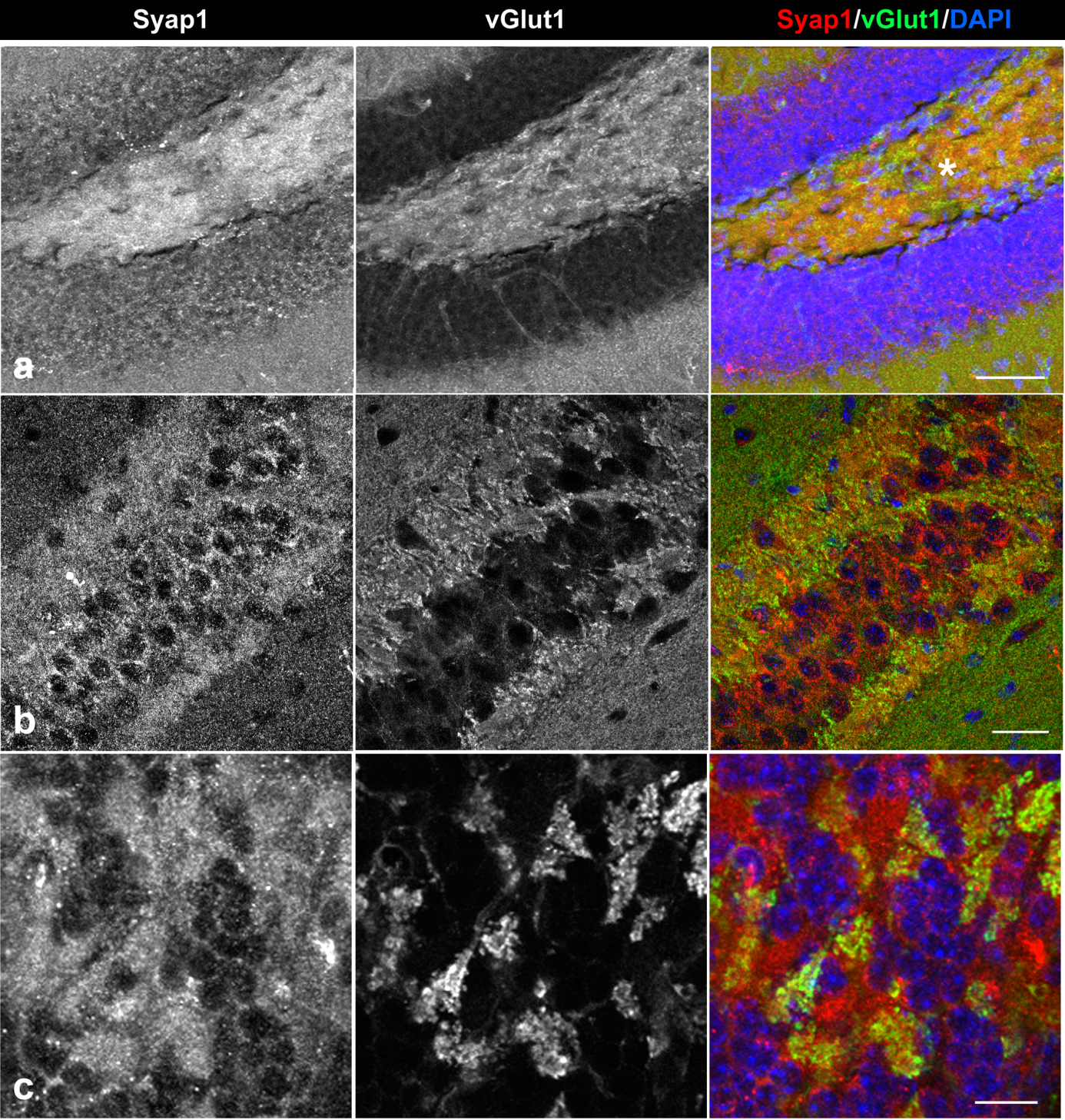
Figure S5

**Fig. S5** Comparison of Syap1 and vGlut1 immunolabeling in the hilus (asterisk in **a**) and the mossy fiber pathways in the CA3 region (**b**) of the hippocampus, as well as the granular layer of the cerebellum (**c**, enlarged cut of Fig. 7c, gray square (main text)). The overlap of Syap1 and vGlut1 immunolabeling indicates the presence of the protein at glutamatergic mossy fiber terminals but Syap1 immunoreactivity is also seen in non-vGlut1-immunoreactive elements. Blue: nuclear DAPI. Scale bars: 50 µm (**a**), 10 µm (**b**, **c**)

**The light microscopical appearance of the Golgi complex is not noticeably altered by Syap1 knock-out**


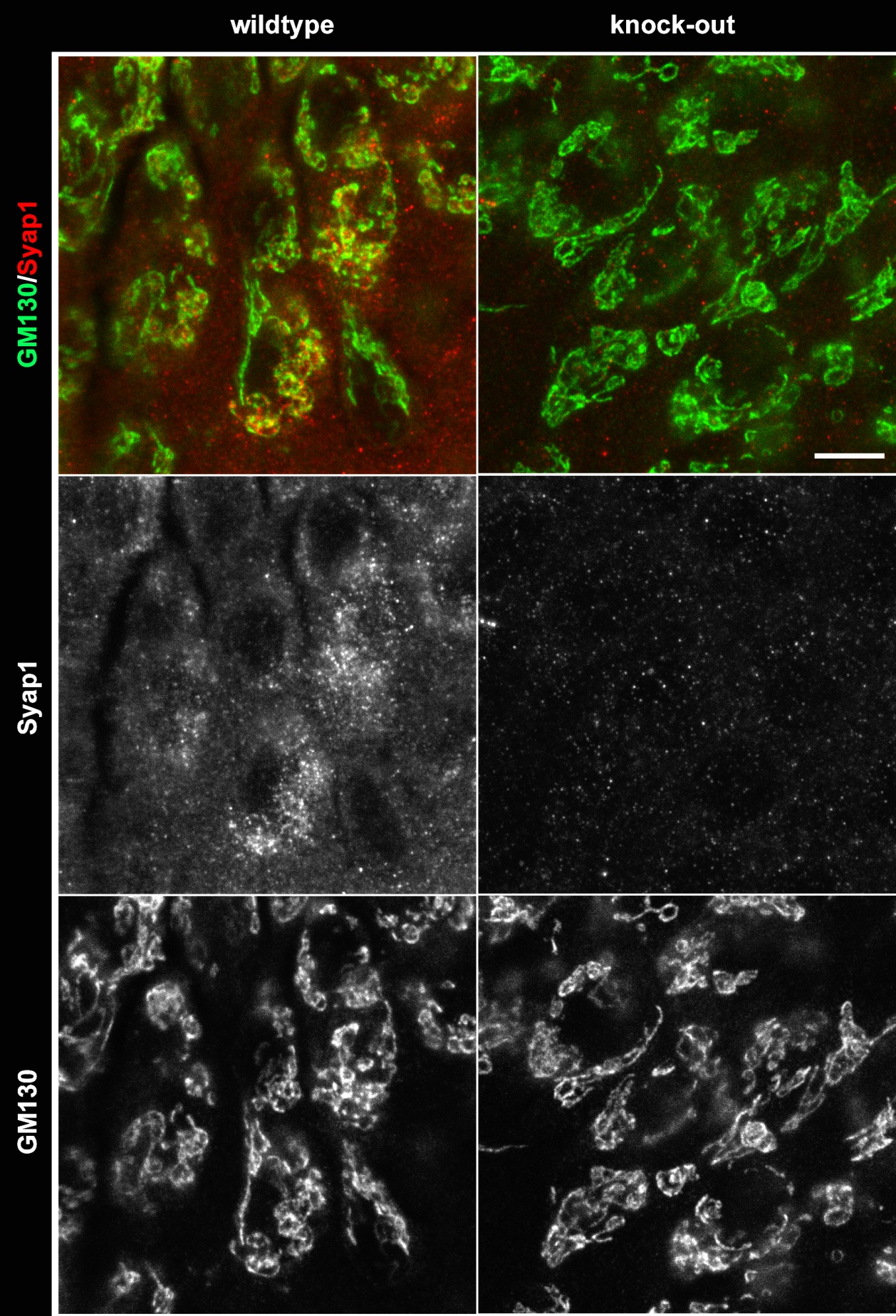


Figure S6

**Fig. S6** High magnification confocal images of perikarya in the hippocampal CA2 region of wildtype and Syap1 knock-out brain sections stained with anti-Syap1 and anti-GM130. No qualitative difference in the GM130 staining is observed, suggesting that loss of Syap1 does not noticeably alter Golgi structures at this resolution (60x, oil). Scale bar 10 µm

**Syap1 is associated with the Golgi complex also in some non-neural cells**

**
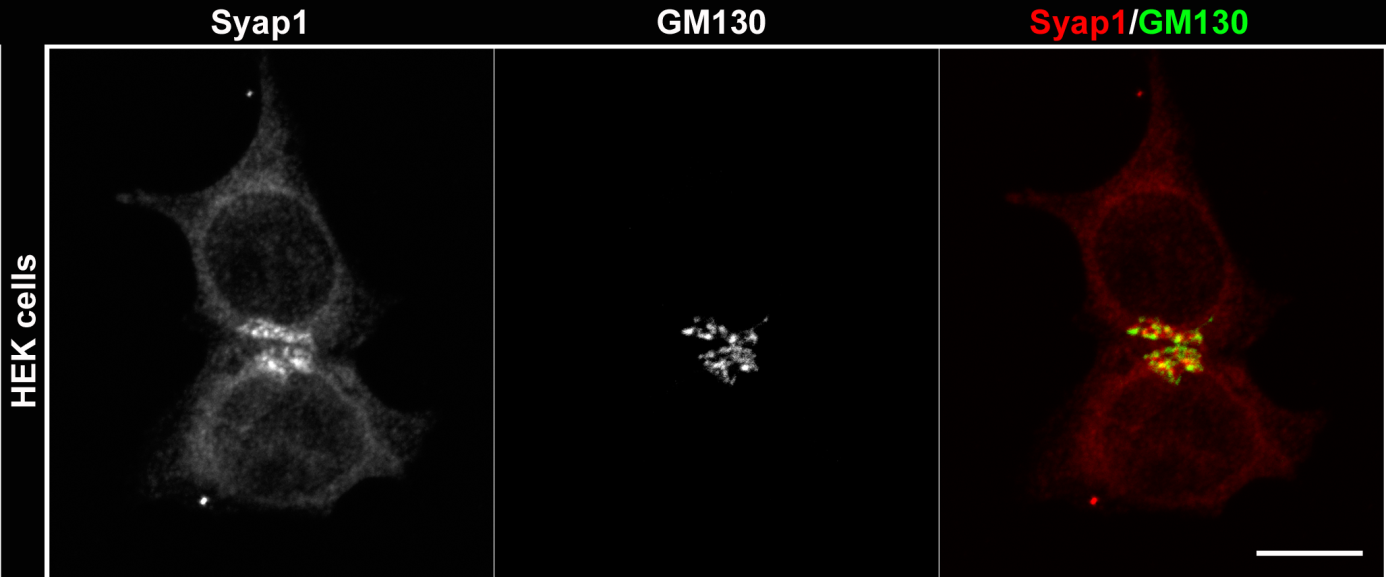
**

Figure S7

**Fig. S7** Image of two human embryonic kidney 293T (HEK) cells labeled with anti-Syap1 and anti-GM130 antisera, showing the close association of the two proteins in a non-neural cell type. Scale bar 10 µm.

**Akt phosphorylation is not reduced in cultured motoneurons upon *Syap1* knock-out (Figs. S8a) or knock-down (Fig. S8b, c, Fig. 9) (quantification).**





Figure S8

Fig. S8 Quantification of pAkt^Thr308^/Akt and pAkt^Ser473^/Akt ratios of BDNF-stimulated or unstimulated Syap1 deficient motoneurons. pAkt^Thr308^/Akt (left) and pAkt^Ser473^/Akt (right) ratios obtained from Western blots as presented in Figs. 13b, c, and d (main text) show no decrease in Akt phosphorylation due to *Syap1* knock-out (a) or knock-down (b, c) under our conditions after two (b) or five (a, c) min of stimulation. Western blot signals were normalized to the stimulated wildtype controls (a) or to the stimulated uninfected control (b, c). Error bars are ±SEM. * = *P* < 0.05; ** = *P* < 0.01; ns = *P* > 0.05





Figure S9

Fig. S9 Akt phosphorylation at Ser^473^ in different compartments of motoneurons is not altered by *Syap1* knock-down. Quantification of pAkt^Ser473^/Akt ratios from immunocytochemical fluorescence of motoneuron preparations as in Fig. 14 (main text) after growth factor starvation and subsequent 5 min BDNF [20 ng/ml] stimulation (a) or under standard growth conditions without starvation and stimulation (b) measured separately for soma, axon, and growth cone. The data support the results obtained by the corresponding Western blot analysis (Fig. 13 (main text), Fig. S8) and fail to reveal compartment-specific differences. The mean ratio for each condition of an experiment was normalized to the corresponding mean of the unstimulated mock control. Error bars are ±SEM. * = *P* < 0.05; ** = *P* < 0.01; ns = *P* > 0.05

**Methods**

**NSC34 cell culture**

The NSC34 cell line is a mouse-mouse neural hybrid cell line, which was developed by fusing aminoptherin-sensitive neuroblastoma cells with motoneuron enriched embryonic spinal cord cells (Cashman *et al.*, 1992). NSC34 cells (CED-CLU140, Biozol, Eching, Germany) were cultured in Dulbecco’s modified Eagle’s medium (DMEM, Invitrogen) supplemented with Glutamax (500 µM), 10% FCS and 1% penicillin /streptomycin at 37 °C under humidified air containing 5% CO_2_. Splitting was done every third day. For antibody specificity test, NSC34 cells were infected with *Syap1* knock-down or mismatch virus in a small volume of medium and cultured for five days before they were lysed in RIPA buffer (50 mM Tris pH 7.4, 150 mM NaCl, 1% Nonidet P-40, 0.1% SDS, 1 mM EDTA and standard protease and phosphatase inhibitors (Roche, Basel, Switzerland, complete mini tablets)).

**Human embryonic 293T (HEK293T) cell culture and SYAP1 overexpression**

To express recombinant Syap1, HEK293T cells (Invitrogen R700-07) were transfected with a FLAG-Syap1 construct via lipofectamine^TM2000^ (Invitrogen). The cells were then plated, cultured for one day and lysed in RIPA buffer. Protein determination was performed with the Bradford protein assay (Biorad, Hercules, CA, USA).

**Table S1** Primer sequences for qRT-PCR and genotyping

| gene | Forward primer | Reverse primer |
| --- | --- | --- |
| *gapdh* | GCAAATTCAACGGCACA | CACCAGTAGACTCCACGAC |
| *Syap1 (Exon 3-4)* | GATGTACCCTGTTGCCCTGG | GCTTAATCAGGGAGATTCGGTAGA |
| *Syap1_2 (Exon 3-4)* | CCAAGAGGATGAGCTACTAAGCAA | GCTTAATCAGGGAGATTCGGTAG |
| *Syap1 (Exon 8-9)* | CGATACGTGCAGCTTAAATCA | TGTAGCTCCTTTTCCCAATCA |
| *Syap1 wildtype* | ACATACACACATGTGTACCACTTT | ACAAGACCACAATTGCAATTTACC |
| *Syap1 knock-out* | ACATACACACATGTGTACCACTTT | GTCCCCCTTCCTATGTAACC |
